# Lactate and Lactylation: What We Know and What We Expect in Tumour Progression

**DOI:** 10.1111/cpr.70293

**Published:** 2026-11-04

**Authors:** Qi Zhang, Zhenzhen Han, Youjia Duan

**Affiliations:** ^1^ Cancer Center, National Center for Infectious Diseases, Beijing Ditan Hospital, Capital Medical University Beijing China; ^2^ Stem Cell and Cancer Center, the First Hospital of Jilin University Changchun China

**Keywords:** cancer therapy, lactate, lactylation, tumour progression

## Abstract

In 2017, lactate was proposed not only as an anaerobic glycolytic metabolite but also as a carbon source involved in the mitochondrial tricarboxylic acid cycle. Lactate plays various biological regulatory roles, such as in energy sources, signalling molecules, and immunomodulatory molecules. Increasing evidence has shown that lactate accumulation in the tumour microenvironment is a hallmark of cancer. However, the mechanisms underlying the role of lactate in cancer progression remain unclear. Recently, various metabolites, including lactate, were shown to regulate epigenetic modifications in a concentration‐dependent manner. In this review, we discuss advancements in the field that show how lactate and lactylation can accelerate the progression of cancer. The synthesis of the studies discussed herein may serve as a theoretical foundation for identifying novel mechanisms underlying rapid tumour progression and provide further insights that will benefit cancer therapies.

## Graphical abstract

Lactate and lactylation in cancer cells have been shown to accelerate tumour development in various ways, including stemness generation, metabolic dysregulation, cell death, chemotherapy resistance, and cell cycle disorder. In addition, lactate and lactylation promote angiogenesis and create an immunosuppressive microenvironment, which promotes tumour proliferation and metastasis.

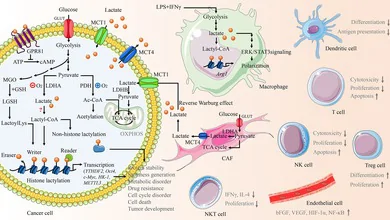

## 1 Introduction

Previously, lactate was deemed a metabolic waste product; however, with the emergence of concepts, such as the Warburg effect and lactate shuttle theory, researchers have realized that “metabolic waste” is an incorrect description [1]. Compared to that in normal tissues, the lactate concentration in cancer tissues can be as high as 10–30 mM and can promote angiogenesis, immune escape, and tumour progression. We therefore hypothesize that lactate is a crucial molecule in malignant cancer development [2, 3].

Metabolism has recently been recognized as a regulator of epigenetic modifications [4]. Studies have shown that multiple metabolites can regulate the structure and function of chromatin and gene transcription via post‐translational modifications, such as S‐adenosylmethionine‐driven methylation, acetyl‐CoA‐driven acetylation, and β‐hydroxybutyrate‐driven butyrylation [4, 5, 6]. Cellular metabolism interacts with the epigenome to remodel chromatin and regulate gene expression, thereby promoting the progression of many diseases, including cancer [4]. This warrants the question: Can lactate also be involved in tumour progression through epigenetic modifications?

In the present study, lactate‐driven lactylation, a novel metabolic epigenetic code, was proposed and speculated to play an important role in cancer development. This review focuses on the conversion of lactate in lactylation and the associated mechanisms in tumour progression. The synthesis of the findings discussed herein may contribute to the identification of novel tumourigenic mechanisms and targets for cancer therapies.

## 2 Lactate in Cancer

### 2.1 Lactate Production and Transport

Pyruvate generated by glucose metabolism can either be converted into acetyl‐CoA and enter the mitochondria for the tricarboxylic acid (TCA) cycle or be catalysed by lactate dehydrogenase (LDH) to generate lactate [7]. For many types of cancer, even in the presence of sufficient oxygen, glycolysis and lactate fermentation are relied upon to generate energy, rather than the comparatively energy‐efficient oxidative phosphorylation, which is called the “Warburg effect” [8]. LDH catalyses the reversible conversion between pyruvate and lactate and consists of two subunits, LDHA and LDHB. Due to its high affinity for pyruvate, LDHA is a key enzyme in glycolysis and lactate production [9, 10]. Elevated LDHA levels in cancer cells create an acidic microenvironment by increasing lactate production, which enhances the number of immunosuppressive cells and decreases that of cytolytic cells, ultimately leading to immunosuppression and immune escape [9].

The communication between lactate and the cell microenvironment relies on monocarboxylate transporters (MCTs). There are four isoforms of MCTs: MCTs 1–4 [11]. MCT1 is ubiquitously expressed in tissues, whereas the other isoforms are tissue‐specific [12]. MCT1 is a passive symporter that transfers lactate along with a proton; MCT2 is enriched in the liver and participates in lactate‐driven gluconeogenesis; MCT3 responds to lactate exchange between the retinal pigment and choroid plexus epithelial cells; and MCT4 has the lowest affinity for lactate but still promotes lactate efflux [13]. Lactate transport also depends on the lactate concentration difference across the membrane, pH, and the levels of other MCT substrates, such as ketone bodies, acetate, pyruvate, and butyrate [14].

MCT1, MCT2, and MCT4 expression has been reported to be upregulated in various cancer cell lines. MCT1 expression is upregulated in oesophageal adenocarcinoma, brain cancer, and cervical cancer; MCT2 expression is upregulated in brain cancer; and MCT4 expression is upregulated in hepatocellular carcinoma, melanoma, breast cancer, and colorectal cancer [13]. MCTs require tight binding to transmembrane glycoproteins to ensure proper folding and transport to the cell surface [15]. For example, MCT1/4 specifically bind to CD147/BASIGIN (BSG), whose expression is upregulated in almost all tumours and is closely related to histological type and tumour progression [15]. BSG knockdown inhibits tumour growth by decreasing MCT1/4 expression and increasing the intracellular lactate level [15]. G protein‐coupled receptor (GPR)‐81, a metabolic sensor, transmits information between lactate and immune cells, forming the lactate–GPR81 signalling pathway that exerts paracrine immunosuppressive functions in macrophages and dendritic cells [16].

Advanced tumours are hypoxic and can secrete large amounts of lactate, which reduces the pH of the microenvironment, thereby maintaining lactate homeostasis and promoting tumour growth and metastasis. Lactate homeostasis is regulated by tissue‐ and function‐specific MCTs [17]. Elevated MCT1 expression in taxol‐resistant breast cancer cells is accompanied by increased glucose metabolism and lactate production. In contrast, after the targeted blockade of MCT1, intracellular lactate accumulation can lead to endogenous cytotoxicity and cell apoptosis [18]. In addition, lactate homeostasis depends on pH, intracellular and extracellular lactate levels, and the concentrations of other MCT substrates such as acetate, pyruvate, and ketones [19].

### 2.2 Lactate in the Tumour Microenvironment

The tumour microenvironment comprises various cells, including cancer cells, immune cells, and cancer‐associated fibroblasts (CAFs), as well as the extracellular matrix, metabolites, and growth factors [1, 20]. Cancer cells and CAFs are the main lactate producers because of their large populations [21]. Cells that predominantly express MCT4 export lactate, whereas MCT1‐rich cells uptake lactate as an energy source and convert it into pyruvate in the TCA cycle to supply energy, which is known as the “reverse Warburg effect” [22].

In principle, tumour cells are recognized as abnormal and are eliminated by immune cells. However, tumour cells can escape immune cells via lactate accumulation [23, 24]. Lactate reduces the intracellular pH in natural killer (NK) cells, leading to mitochondrial dysfunction and apoptosis [25]. Natural killer T (NKT) cells are immune cells that exert anti‐tumour activity. Lactate prevents NKT cells from secreting interferon‐γ and interleukin (IL)‐4 and interferes with NKT cell activation by blocking mTOR signalling [2]. High lactate concentrations impair CD8+ T cell function and can induce NK cell apoptosis [24, 26]. Lactate has also been reported to induce PD‐L1 expression on neutrophils via the MCT1/NF‐κB/COX‐2 pathway, thereby suppressing T cell cytotoxicity [27].

Lactate not only induces vascular endothelial growth factor (VEGF) and arginase 1 (Arg1) expression via HIF‐1α but also activates ERK/STAT3 signalling in breast cancer, ultimately promoting macrophage polarization towards the M2 type [22, 26, 28]. CCL17, a chemokine secreted by lactate‐induced M2 macrophages, suppresses dendritic cell maturation, limits antigen presentation, and recruits regulatory T cells (Treg cells), thereby promoting the invasion of pituitary adenoma cells [29]. Once nutrient deficient, Treg cells utilise lactate to proliferate and enhance immunosuppressive function [30]. Aside from the HIF‐1α pathway, lactate inhibits the transcription factor EB (TFEB)‐mediated expression of the macrophage‐specific vacuolar ATPase subunit ATP6V0d2 in an mTORC1‐dependent manner. Upregulated HIF‐2α levels, targeted by ATP6V0d2, then promote tumour growth and metastasis by activating the transcription of VEGF and Arg1 [31]. In contrast, various studies have proposed that lactate enhances Toll‐like receptor 4 signalling, the NF‐κB‐dependent transcription of inflammatory genes, and the pro‐inflammatory effects of macrophages [32, 33].

Generally, lactate‐driven immune evasion occurs through GPR81 binding via autocrine (tumour cells) and paracrine (immune cells, endothelial cells, and adipocytes) pathways [20, 34]. After the activation of GPR81, cAMP and protein kinase A signalling are inhibited, pro‐inflammatory cytokine production is reduced, and the activity of regulatory factors, such as IL‐10, is induced [35]. Furthermore, lactate induces VEGF and basic fibroblast growth factor activity and stimulates NF‐κB/IL‐8 signalling by activating the HIF‐1α pathway to encourage angiogenesis in vascular endothelial cells [24, 35]. Lactate also induces hyaluronic acid expression in the matrix surrounding cancer cells, thereby increasing cancer cell motility and angiogenesis [36]. Lactate therefore promotes immune inhibition and escape by suppressing the activation and proliferation of NK, NKT, and CD8+ T cells; inducing Treg proliferation and the M2‐type polarization of macrophages; and regulating immune factors, ultimately leading to tumour progression [35, 37].

### 2.3 Lactate in Tumour Progression

In addition to serving as an energy source and promoting immunosuppression, lactate drives tumour progression by regulating stemness and genetic modifications. Lactate has been reported to cause a 10‐fold increase in tumour metastasis, suggesting that lactate may promote cancer cell stemness [8]. Transcription analyses have further shown that lactate increases the transcriptional levels of stemness‐related genes, which are key to cancer stem cell (CSC) generation, tumour recurrence, and metastasis [8, 38, 39].

Lactate has further been reported to enhance the motility and invasiveness of breast cancer cells by increasing ERK1/2 phosphorylation and reducing E‐cadherin expression [40]. Notably, lactate, an HDAC inhibitor, has been found to accelerate tumour progression via acetylation and acetylation‐related gene expression [4, 38]. These findings are among the first to link lactate to metabolism and epigenetics.

## 3 The Discovery of Lactylation

In 2019, Di Zhang et al. described an unknown histone modification, lactylation, and used isotope‐labelled lactate for metabolic tracing to demonstrate that lactate contributed to histone lactylation. In addition, hypoxia, M1 macrophage polarization, exogenous glucose, and the inhibitor rotenone, which are beneficial for glycolysis, were found to promote lactylation, which was attenuated by the inhibition of glycolysis [41].

### 3.1 Substrate

The discovery of lactyl‐CoA revealed a transition from lactate to lactylation. Lactyl‐CoA is less abundant than the common acetyl‐CoA [42]. Histone lysine lactylation occurs after acetylation during M1 polarization [41, 43] and may be determined by the concentration of acyl‐CoA and lactyl‐CoA [44]. In addition, lactate can be directly added as a substrate to proteins with the assistance of alanyl‐tRNA synthetase 1 (AARS1) and AARS2 [45].

Non‐enzymatic lysine lactoylation has been previously reported [46]. Glutathione (GSH) and methylglyoxal produced by glycolysis have been found to be catalysed by glyoxalase 1 to produce lactoylglutathione (LGSH). The latter is then converted to GSH and D‐lactate by glyoxalase 2. LGSH is the core of non‐enzymatic acyl transfer, that is, the lactate moiety from LGSH to protein Lys residues, thus forming the “LactoylLys” modification.

### 3.2 Writers, Erasers, and Readers

Initially, Zhang et al. proposed that a potential candidate for lactylation is the acetyltransferase p300 [41]. During cell differentiation, p300‐mediated lactylation and acetylation are overexpressed in the promoter regions of pluripotency genes, such as *Oct4*, *Sall4*, and *cMyc* [47]. The depletion of p300 in HCT116 and HEK293T cells significantly reduces histone lactylation levels [47, 48]. TIP60, a member of the MYST subfamily of histone acetyltransferases, participates in DNA repair by regulating the lactylation of NBS1 [49]. AARS1 and AARS2 directly use lactate as a substrate to modify the lactylation of proteins [45, 50, 51]. AARS1 directly senses lactate and mediates the lactylation of the DNA‐binding region of the tumour suppressor P53 in an ATP‐dependent manner. Lactylated P53 cannot bind to DNA to exert its tumour suppressor function, leading to tumour progression [50]. AARS1 lactylates the YAP pathway to promote the progression of gastric cancer [51]. Other lactyltransferases include HAT1, KAT2A (GCN5), KAT8 (MOF), ME‐PCT, and RE‐PCT [52, 53, 54, 55, 56, 57].

Class I histone deacetylases, including HDAC1–3, are lysine delactylases, among which HDAC3 is the most efficient eraser, and HDAC1 and HDAC3 are site‐specific [58]. HDAC1–3 and p300 exhibit tumourigenic abilities by regulating lactylation in various cancers, such as leukaemia and prostate, gastric, and colorectal cancer [59, 60, 61, 62]. The expression of HDAC1–3 and p300 in liver cancer is related to B cell and M2 macrophage infiltration [63]. SIRT3, another deacetylase, has been reported to exert a slight delactylation effect and delactylates non‐histone cyclin E2 to inhibit hepatocellular carcinoma progression [64]. SIRT3 is also a specific eraser of H3K9 lactylation in oesophageal squamous cell carcinoma [65]. Other erasers have been confirmed, including SIRT1 and SIRT2 [66]. In gastric cancer cells with high SIRT1 expression, the lactylation of H3K18 at the *H19* promoter is significantly reduced, thereby leading to decreased H19 expression. The inhibition of H19 weakens LDHA and H3K18 lactylation levels [67]. SIRT6 also exhibits delactylase activity, particularly at H3K9 and H3K18 sites [68].

DPF2 may be an effector of H3K14 lactylation in cervical cancer. DPF2 has been reported to potentially recognize the lactylation of H3K14 through its double PHD fingers and co‐localize with lactylated H3K14 at the promoters of oncogenes to activate gene transcription [69]. Some studies have shown that Brg1 may recognize H3K18 lactylation at the promoters of various genes related to stemness and epithelial adhesion during the Dux‐mediated reprogramming of induced pluripotent stem cells [70, 71].

## 4 Lactylation in Cancer

### 4.1 Lactylation in the Tumour Microenvironment

Studies on the correlation between lactylation and Arg1 in tumour‐associated macrophages (TAMs) have highlighted the role of lactylation in tumour immunity [41, 72]. Researchers have detected a positive correlation between histone lactylation and Arg1 by reparative M2 in TAMs isolated from B16F10 melanoma and LLC1 lung tumours, independent of acetylation [4, 5, 41]. Macrophages with high Arg1 expression have been found to co‐express PD‐L1 and Trem2 to protect tumour cells from inflammation and are thus called regulatory macrophages [73, 74]. We speculate that histone lactylation is an important factor in the transition from macrophages to regulatory macrophages via Arg1 expression. Regulatory macrophages further inhibit polyclonal T cells to promote tumour growth [73, 75]. Lactylation is also involved in the regulation of macrophage polarization in cervical cancer. Lactate secreted by cervical cancer cells induces macrophage polarization towards the M2 type by promoting the expression of GPD2 [76]. In cervical cancer, the small‐molecule protein ICAT activates the c‐Myc–ENO1–glycolysis pathway, upregulates H3K18 lactylation and Arg1 expression, and induces M2 macrophage expression [77]. Similar lactylation‐dependent M2 macrophage polarization occurs in glioma and bladder cancer [78, 79]. The factors secreted by glioblastomas promote high glycolysis and lactate production of monocyte‐derived macrophages, further inducing the expression of IL‐10 and inhibiting T cell activity [80].

Vascular cell adhesion molecule 1 (VCAM1) participates in the regulation of inflammatory responses. In gastric cancer, high‐level lactylation promotes the expression of VCAM1, which increases the expression of the chemokine CXCL1 in an AKT–mTOR1 pathway‐dependent manner. This leads to epithelial–mesenchymal transition and the recruitment of M2 macrophages, thereby promoting immunosuppression and the progression of gastric cancer [81]. High H3K18 lactylation in pancreatic cancer can activate the transcription of acetyl‐CoA acetyltransferase (ACAT2), which can acetylate mitochondrial carrier homologue 2 to stabilize its expression and disrupt oxidative phosphorylation, thereby promoting lactate production and the reactivation of ACAT2. This positive feedback loop assists in the secretion of cholesterol by small extracellular vesicles and promotes the polarization of macrophages to the M2 type [82].

Lactate has also been reported to increase membrane‐organizing extension spike protein (MOESIN) lactylation at Lys72 in Treg cells. Lactylated MOESIN interacts with transforming growth factor β receptor I to activate transforming growth factor β and downstream SMAD3 signalling. Eventually, the forkhead box P3 (FOXP3) transcription factor is expressed at high levels to maintain the immunosuppressive functions of Treg cells [83]. Clinically, lower levels of MOESIN lactylation in Treg cells were observed in patients responding to anti‐PD‐1 therapy than in nonresponders, suggesting that elevated MOESIN lactylation may be associated with an immunosuppressive Treg phenotype and reduced responsiveness to PD‐1 blockade [83]. Compared with anti‐PD‐1 treatment alone, the combination of anti‐PD‐1 and LDH inhibitors has a stronger anti‐tumour effect [84]. In Treg cells with malignant pleural effusion (MPE), a high level of lactate can gradually enhance the expression of NF‐κB p65 and tumour necrosis factor receptor 2 (TNFR2) by promoting the H3K18 lactylation of NF‐κB p65, thereby increasing the immunosuppressive effects of Treg cells and malignancy of the tumour [85]. TNFR2+ Treg cells can also be recruited by lactate and the chemokine ligand CXCL16 secreted by CAFs and play an immunosuppressive role in MPE [86].

Similar to Treg cells, a small population of NKT cells expressing FOXP3 appear to promote tumour progression rather than exert anti‐tumour activity. For example, in hepatocellular carcinoma, the proportion of FOXP3^+^ NKT cells is high, which affects clinical outcomes [87]. In MPE, glycolysis and the pyruvate metabolism of FOXP3^+^ NKT cells are increased, as is the expression of MCT1 and LDHB. Increased lactate uptake and production upregulate FOXP3 expression by promoting the histone lactylation of the *FOXP3* promoter, eventually generating a tumour‐promoting immune environment [88].

Some studies have suggested that the lactylation of PD‐L1 is one of the mechanisms underlying immune escape in the anti‐tumour response of cytotoxic T cells [89]. STAT5 promotes PD‐L1 expression by facilitating histone lactylation, thereby inhibiting T cell activation and driving immunosuppression in acute myeloid leukaemia [90, 91]. In non‐small cell lung cancer, H3K18 lactylation activates the pore membrane protein 121, which further promotes the binding of MYC to the *CD274* promoter, inducing the expression of PD‐L1 and immunosuppression [92]. The highly expressed claudin‐9 in gastric cancer further activates glycolysis through the PI3K–AKT–HIF‐1α pathway, promoting the lactylation of PD‐L1 and inhibiting the anti‐tumour activity of CD8+ T cells [93]. In head and neck squamous cell carcinoma, high levels of H3K9 lactylation promote the expression of IL‐11, which then activates the JAK2–STAT3 pathway to enhance the expression of immune checkpoint genes and cause the dysregulation of CD8+ T cells [94]. The combination of glycolysis inhibitors and anti‐PD‐1 antibodies could be considered a new direction for cancer treatments that restore the anti‐tumour activity of CD8+ T cells [92].

Methyltransferase‐like 3 (METTL3), the core of the MTase complex that promotes m6A modifications in tumour‐infiltrating myeloid cells, is associated with immunosuppression and poor prognoses in colon cancer [95]. The acidic microenvironment of colorectal cancer induces the H3K18 lactylation of *METTL3* to upregulate METTL3 expression, which contributes to the translation of *Jak1* mRNA by reinforcing the m6A modification of *Jak1* mRNA. The downstream STAT3 signal is activated to increase the expression of certain factors, such as IL‐6, IL‐10, and iNOS. Eventually, immunomodulatory factors induced by the METTL3–m6A–Jak1–STAT3 axis strengthen immunosuppression and promote colon cancer growth [95].

Researchers have attempted to link lactylation with immunotherapy. One study proposed that LDHA inhibitors can assist in CAR‐T cell anti‐tumour therapy. The LDHA inhibitor oxamate was found to reduce the lactylation of H3K18 in the tumour microenvironment, thereby decreasing the number of CAR‐Treg cells and activity of the gene promoters of *CD39*, *CD73*, and *CCR8*, which led to the reprogramming of the glucose metabolism of glioblastoma multiforme stem cells and activation of tumour immunity [96, 97].

Angiogenesis is crucial for tumour progression, as it provides oxygen and nutritional support for cell survival. In melanoma, lactate and lactylation mediate the transcriptional activity of suppression of tumourigenicity 2 in endothelial cells, thereby increasing the sensitivity of endothelial cells to IL‐33, which has pro‐angiogenic properties [98]. In prostate cancer, lactate upregulates HIF‐1α lactylation to stabilize HIF‐1α, even in the presence of sufficient oxygen. HIF‐1α can then promote angiogenesis and vasculogenic mimicry by enhancing KIAA1199 expression, a hyaluronic acid binding protein [99]. Lactylation therefore enables the acidic microenvironment to regulate the activity of various immune cells and immune factors and promote angiogenesis, providing a favourable microenvironment for tumour growth (Figure 1).

**FIGURE 1:**
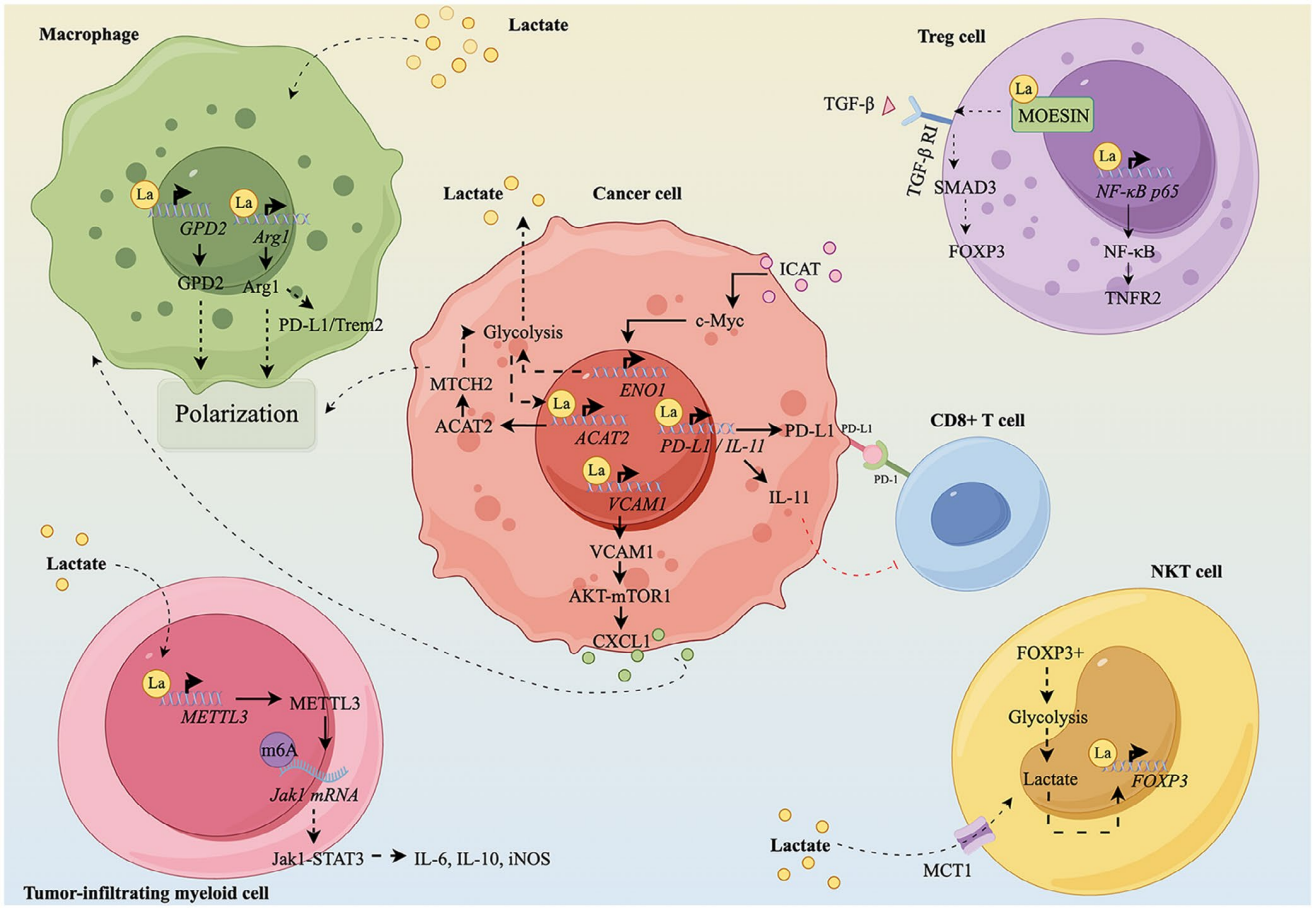
Lactylation in tumour microenvironment. The tumour microenvironment is composed of various immune cells, extracellular matrix, and blood vessels. Lactylation modification promotes tumour progression by regulating various immune cells and factors to create an immunosuppressive microenvironment (by Figdraw).

### 4.2 Lactylation in Tumour Progression

#### 4.2.1 Lactylation and Oncogene Expression

In addition to its metabolic role, lactate is involved in tumour progression as a signalling molecule or pro‐inflammatory factor [19]. Furthermore, elevated lactate levels under highly glycolytic conditions can act as epigenetic regulators that drive downstream oncogene expression [41]. YTH N6‐methyladenosine RNA‐binding protein 2 (YTHDF2), an m6A reader, usually recognizes m6A in the 3′‐UTR of mRNA and decreases mRNA stability; additionally, it has been confirmed to play a critical role in the progression of several tumours [100, 101]. Lactylation is highly expressed in human ocular melanoma and is negatively correlated with clinical outcomes [100]. Mechanistically, the lactylation of H3K18 in the *YTHDF2* promoter region activates YTHDF2 expression. Subsequently, YTHDF2 recognizes m6A in the 3′‐UTR of *PER1* and *TP53* mRNA, two tumour suppressor genes, and facilitates their degradation, which accelerates ocular melanoma proliferation, migration, and clone formation [100]. Similarly, H3K18 lactylation‐mediated upregulation of YTHDF2 promotes stemness in non‐small cell lung cancer by reducing the expression of the tumour suppressor secreted frizzled‐related protein 2 [102].

In colon cancer cells, lipopolysaccharide promotes *LINC00152* expression by inducing the H4K8 lactylation of its promoter, thereby inhibiting inflammatory responses and promoting cancer cell migration and invasion [103]. In breast cancer, lactate increases the H3K18 lactylation of the oncogene *c‐Myc* promoter to upregulate c‐Myc expression, which subsequently promotes serine/arginine splicing factor 10 to drive the alternative splicing of *MDM4* and *Bcl‐x*, ultimately promoting tumour progression [104]. The oncogene *DCBLD1* (discoidin, CUB, and LCCL domain‐containing Type I) promotes tumour cell invasion and metastasis in cervical cancer. Lactate further activates HIF‐1α, promotes the enrichment of HIF‐1α in the promoter region of *DCBLD1*, and drives the lactylation of DCBLD1 K172. Highly expressed DCBLD1 activates the pentose phosphate pathway to accelerate cervical cancer progression in a glucose‐6‐phosphate dehydrogenase (G6PD)‐dependent manner [105].

#### 4.2.2 Lactylation and Metabolic Maladjustment

Lactylation shows a preference for enzymes involved in various metabolic pathways, including the TCA cycle and carbohydrate, amino acid, fatty acid, and nucleotide metabolism [106]. In non‐small cell lung cancer, lactate decreases the expression of glycolysis‐related enzymes, such as hexokinase‐1 and pyruvate kinase, while upregulating the expression of the TCA cycle‐related enzymes succinate dehydrogenase and isocitrate dehydrogenase via histone lactylation. This weakens glucose uptake and glycolysis, leading to metabolic disorders [107, 108]. The TCA cycle is an oxygen‐dependent metabolic pathway. In renal cell carcinoma, the malate dehydrogenase 2 (MDH2) of the TCA cycle becomes a lactylation‐targeting site in the low‐glucose and high‐lactate microenvironment. When the K239 site of MDH2 undergoes lactylation, the enzymatic activity of MDH2 increases, promoting the generation of ATP and NADPH, alleviating oxidative stress, and maintaining mitochondrial function, thereby facilitating the progression of renal cell carcinoma [57]. The lactylation of G6PD activates the pentose phosphate pathway, which enables the interaction between glycolysis and the pentose phosphate pathway to promote tumour progression [109].

In lenvatinib‐resistant hepatocellular carcinoma, increased lactylation levels of the m6A‐reader IGF2BP3 promote the expression of phosphoenolpyruvate carboxykinase (PCK2). As a key enzyme in gluconeogenesis, PCK2 provides substrates for serine synthesis and drives the reprogramming of serine metabolism [110, 111]. Pyrrolidine‐5‐carboxylic acid reductase 1 (PYCR1) is a key enzyme in proline metabolism and is highly expressed in various tumours, including bladder cancer. Hypoxia promotes the expression of PYCR1 and accelerates glycolysis and lactate accumulation. The lactate‐driven H3K18 lactylation of the glutamine transporter gene *SLC6A14* promotes its transcription and drives glutamine catabolism, leading to the proliferation and metastasis of bladder cancer cells [112]. In colorectal cancers with KRAS mutations, the high expression of lactate and lactylation modification of H3K9 can promote the transcriptional expression of the cholesterol transporter GRAM domain‐containing protein 1A, after which cholesterol metabolism can drive tumour progression [113].

Analogous metabolic disorders occur in hepatocellular carcinoma. Lactylated proteins in tumour tissues are associated with almost all dysregulated metabolic pathways, especially the degradation of fatty acids and branched amino acids. The upregulated lactylation of branched amino acid degradation pathways may be associated with tumour progression. The pathogenic characteristics of clinical samples have consistently been associated with the upregulation of lactylated proteins in various metabolic pathways. For example, increased AFP, tumour thrombus, and tumour diameters are related to the amino acid synthesis, glycolysis, and spliceosome pathways, respectively [106]. Adenylate kinase (AK2), a key enzyme in energy metabolism, is involved in the reversible transfer of the terminal phosphate group from ATP to AMP, generating ADP [114]. A previous study showed that the tumour, along with high levels of AK2‐K28 lactylation, was accompanied by the downregulation of drug and retinol metabolism and upregulation of DNA replication and nucleotide excision repair processes. In addition, tumours with high AK2‐K28 lactylation showed elevated lactylation levels in gluconeogenesis, amino acid synthesis, fatty acid metabolism, and antigen presentation, all of which contribute to malignancy in hepatocellular carcinoma [106].

#### 4.2.3 Lactylation and Cell Cycle Disorder

Metabolic differences are closely associated with the cell cycle. In HeLa cells, G1 phase glucose metabolism is mediated by the TCA cycle, whereas the S phase is dependent on glycolysis [115]. When investigating the role of the cell cycle in various biological processes, Lin et al. found that some lactylated proteins were involved in regulating the cell cycle. The authors observed that some non‐histones, such as 14–3‐3 protein gamma/epsilon/zeta and high‐mobility group protein B1, had lactylation sites [116]. Cyclin E2, which controls the G1/S transition, has further been found to promote the proliferation, invasion, and migration of hepatocellular carcinoma cells via lactylation of K348 [64]. Royal jelly acid, a major unsaturated fatty acid, induces S‐phase cell‐cycle arrest in liver cancer cells and interferes with cell proliferation by reducing lactate production and inhibiting the lactylation of H3K9 and H3K14 [117].

Cell cycle dysregulation leads to chemotherapy resistance. Pemetrexed resistance in lung cancer‐driven brain metastasis is associated with the aldo‐keto reductase family 1 member B10, which promotes LDHA expression and lactate production. This leads to the increased H4K12 lactylation of the cell cycle‐associated gene *CCNB1* promoter, thus accelerating DNA replication and cell cycle, leading to acquired resistance to pemetrexed [118]. The discovery of lactylation in cell cycle‐related proteins and genes may further inform the correlation between the cell cycle and tumour progression and aid in the development of cell cycle‐based cancer therapies.

#### 4.2.4 Lactylation and Self‐Renewal Ability

Self‐renewal is a defining characteristic of stem cells and CSCs and is generally regulated by epigenetic and metabolic networks [119, 120]. Gli‐like transcription factor 1, a reprogramming factor, activates glycolysis and increases lactate levels, which enrich lactylation at the promoters of the pluripotency factors *Oct4*, *Sall4*, and *Mycn*, ultimately inducing pluripotency [47]. Similar to stem cells, CSCs possess self‐renewal ability and tumourigenicity. CSCs upregulate H3K9 and H3K56 lactylation to induce tumourigenicity in hepatocellular carcinoma, which is suppressed by demethylzeylasteral, a triterpene anti‐tumour compound [121]. H3K18 and H4K12 in colon cancer can contribute to stemness and CSC progression [122, 123]. High levels of glycolysis in CSCs promote the lactylation of aldolase A (ALDOA) at K230/322, which precisely inhibits tight binding between ALDOA and DDX17, thus strengthening the regulatory role of DDX17 in the self‐renewal ability of stem cells [124]. Lactate also induces the K33 lactylation of TWIST1 in liver cancer cells, facilitating its transport into the nucleus and regulating the expression of genes involved in epithelial–mesenchymal transition, including the upregulation of *MYC* and *SNAIL2* and the downregulation of *CDH1* [125].

Several studies have shown that the Wnt/β‐catenin signalling pathway is one of the key signals in maintaining CSC self‐renewal [126]. Further studies have shown β‐catenin is associated with various biological behaviours, such as cell proliferation, differentiation, and oncogenicity [127]. Recently, β‐catenin was shown to be highly expressed in hypoxic colorectal cancer and to promote cancer cell growth and stemness. Mechanistically, hypoxia induces the lactylation of β‐catenin to enhance its protein stability and encourages β‐catenin‐mediated malignant behaviour. Once *β‐catenin* is knocked down, hypoxia‐induced colorectal cancer cell proliferation and stemness generation can be prevented, which can be rescued by agonists of the Wnt pathway [127]. Hypoxia also induces the lactylation of stemness genes and metabolic enzymes to generate stemness and accelerate tumour progression in colon, non‐small cell lung, and oesophageal cancers [123, 128].

#### 4.2.5 Lactylation and Cell Death

SLFN5, an apoptosis‐promoting factor, is inhibited in triple‐negative breast cancer by high levels of lactate and H4K12 lactylation. This reduces the activity of the *SLFN5* promoter and transcription, thereby inhibiting the subsequent apoptotic pathway and increasing the malignancy of the tumour [129]. Unlike apoptosis, new forms of cell death, such as ferroptosis and cuproptosis, have been discovered and confirmed to be closely related to tumour development. The former is a death mode driven by iron‐dependent lipid peroxidation, whereas the latter is mitochondrial‐dependent cell death induced by copper ions [130, 131]. The lactylation of H4K12 in colorectal CSCs inhibits ferroptosis by promoting the expression of the downstream gene *GCLC* (glutamate‐cysteine ligase catalytic subunit), ultimately promoting tumour progression [122]. Cancer cells with *KRAS* mutations successively activate the MEK/ERK and LDHA/lactate signalling pathways, promoting the lactylation of glutamate‐cysteine ligase modifier (GCLM), resulting in an increase in GSH, a reduction in lipid peroxidation, and resistance to ferroptosis [132]. Lactate and lactylation in the tumour microenvironment also make tumour cells resistant to ferroptosis. The acidic microenvironment constructed by CAFs is associated with elevated lactylation modifications of triple‐negative breast cancer cells, promoting the high expression of ZFP64 and inhibiting ferroptosis, leading to an increase in resistance to doxorubicin [133]. Ferroptosis also occurs in immune cells, and the lactylation of ACSL3 in TAMs can enhance ferroptosis resistance and maintain M2 polarization, thereby promoting the progression of colorectal cancer [134]. In addition, the lactate–IGF2BP2–Nrf2–GPX4 and PRMT5 K240la–ALKBH5–SLC7A11 axes in colorectal cancer, L‐lactate/PSMD14/ENO1 axis in cholangiocarcinoma, GLUT3–H3K18la–MAPKAP1 axis in gastric cancer peritoneal metastasis, β‐catenin K193la–TCF4–GPX4 axis in non‐small cell lung cancer, and H3K18la–TRA2A–STIL axis in ovarian cancer all contribute to ferroptosis resistance and the establishment of antioxidant defence mechanisms [135, 136, 137, 138, 139, 140, 141].

Lactylation is also linked to glycolysis and cuproptosis in gastric cancer. When tumour cells are subjected to copper stress, AARS1/2 promotes the lactylation of the K229 site of METTL16. Lactylated METTL16 upregulates the expression of the cuproptosis regulator FDX1 through the m6A modification, thereby driving cuproptosis [53]. NAT10 catalyses the N4‐acetylcytidine modification (ac4C). The lactylation of NAT10 enhances its catalytic activity, thereby catalysing the ac4C modification of the downstream cuproptosis‐related *DLAT* mRNA, promoting the expression of DLAT, and inducing cuproptosis in colorectal cancer cells, which can be reversed by SIRT1 [142]. These findings highlight a potential tumour‐suppressive role of lactylation by promoting cuproptosis through substrate‐specific regulation of METTL16 and NAT10.

Lactylation‐related autophagy promotes tumour progression. ULK1, one of the most important protein kinases in autophagy, induces the phosphorylation of LDHA to increase its enzymatic activity, which leads to lactate production and the lactylation of proteins such as Vps34. Vps34 is a key regulator of autophagy, and the lactylation of Vps34 in lung and gastric cancers can promote autophagy in cancer cells to accelerate tumour progression [143, 144]. As an autophagy enhancer, RUBCNL acts by promoting autophagosome maturation in colorectal cancer cells treated with bevacizumab. Mechanistically, after bevacizumab treatment, glycolysis and lactate production increase, promoting the H3K18 lactylation at the *RUBCNL* promoter. RUBCNL recruits the PtdIns3K complex to promote autophagosome maturation, autophagy, cancer progression, and bevacizumab resistance [145]. The transcription factor TFEB is also closely associated with lysosome formation and autophagy. In pancreatic cancer, high levels of glycolysis promote the K91 lactylation of TFEB, inhibit its ubiquitination and degradation, and lead to increased TFEB stability, thereby promoting the expression of genes related to lysosome formation and autophagy [146]. Pyroptosis, a pro‐inflammatory type of programmed cell death, has also been shown to be associated with lactylation [130, 131]. Mannose has an inhibitory effect on bladder cancer. Mechanistically, mannose binds to the catalytic enzyme PKM2 during glycolysis, reducing the catalytic activity of PKM2 and inhibiting lactate production, which further reduces the lactylation of PKM2‐K433 and promotes the translocation of PKM2 to the cell nucleus. Eventually, the NF‐κB pathway is activated, and NLRP1/Caspase‐1/GSDMD/IL‐1β‐dependent pyroptosis is induced. This process simultaneously promotes an anti‐tumour immune response and enhances the efficacy of immune checkpoint inhibitors [147].

#### 4.2.6 Lactylation and Chemotherapeutic Resistance

Most chemotherapeutic drugs damage the DNA of tumour cells. The rapid repair of tumour cell DNA is a key factor in drug resistance. The lactylated DNA repair‐related protein, NBS1, forms an MRN complex with MRE11 and RAD50. The purpose is to sense double‐strand break sites and promote the accumulation of homologous recombination repair proteins, ultimately activating the DNA damage repair pathway [49]. As a homologous recombinant protein, lactylated MRE11 promotes resistance to chemotherapy and radiotherapy in a similar way [148, 149]. The lactylation of RAD51 has further been confirmed to be involved in homologous recombination and cisplatin resistance in ovarian cancer [52]. During the cisplatin‐resistant stage of triple‐negative breast cancer, the lactylation of METTL3 is significantly decreased, which is mediated by HDAC2. Delactylated METTL3 forms a methyltransferase complex and promotes the m6A modification of DNA damage repair‐related genes, thereby exerting resistance [150]. Taken together, these studies highlight the highly context‐dependent effects of lactylation in cancer. Whereas lactylation of METTL16 and NAT10 promotes cuproptosis, lactylation of DNA repair proteins such as NBS1, MRE11, and RAD51 can facilitate DNA damage repair and therapeutic resistance, while METTL3 delactylation similarly contributes to chemoresistance. Thus, the biological consequences of lactylation depend on the specific substrate and cellular context, and broad inhibition of lactylation cannot be assumed to be uniformly beneficial.

Once the transcription factor YY1 is abnormally activated, it promotes chemotherapy resistance in various tumours by upregulating DNA repair and multidrug resistance genes [151]. YY1 in CAFs is involved in the construction of the CSC niche associated with exosomal miR‐146a‐5p, thereby increasing cisplatin resistance in bladder cancer [151, 152]. Lactylation‐driven YY1 has been found to interact with the cisplatin resistance driver factor HNRNPU to regulate cisplatin resistance in bladder cancer [151]. Lactate secreted by CAFs has further been reported to promote the lactylation of K453 in ANTXR1 in colorectal cancer cells, activate the RhoC/ROCK1/SMAD5 signalling pathway, and induce stemness and oxaliplatin resistance [153]. Concurrently, some lactylation‐related genes have been associated with chemotherapy resistance in platinum‐resistant ovarian cancer, such as *ALDH1A1* and *S100A4* [154].

#### 4.2.7 Lactylation and Other Modifications

Lactylation may interact with ubiquitination in the lactylation‐pro‐cancer process. Lactylation and ubiquitination jointly contribute to hepatocellular carcinoma progression [155]. Recent studies have revealed an association between lactylation and m5C modifications. The lactylation at K508 of the methyltransferase NSUN2 enhances its catalytic activity. Subsequently, lactylated NSUN2 targets the *GCLC* mRNA and promotes m5C modification and mRNA stability. Activated GCLC further inhibits lipid peroxidation and protects cancer cells from doxorubicin‐induced ferroptosis by inducing GSH synthesis [156]. Similarly, in colorectal cancer, the H3K18 lactylation of *NSUN2* and lactylation at K356 of NSUN2 enhance NSUN2 activity and mediate m5C modifications, which promote glucose metabolism reprogramming by targeting *ENO1* [157].

Lactylation regulates m6A‐related pathways to promote immunosuppression and tumour development [95, 100, 158]. RBM15, an m6A writer, regulates overall m6A levels by binding to the METTL3–WTAP complex. In bladder cancer, hypoxia promotes RBM15 expression through H3K18 lactylation. RBM15 induces the m6A modification of *IGFBP3* mRNA and upregulates IGFBP3 expression. Eventually, IGFBP3 complexes with p‐EGFR and p‐DNA‐PKcs and promotes their nuclear translocation, thereby enhancing DNA repair and cisplatin resistance [159, 160].

The mechanisms by which lactylation accelerates tumour progression are diverse and mainly divided into histone‐ and non‐histone‐mediated pathways according to the position of the modification (Figure 2). Histone‐mediated lactylation primarily affects gene expression, whereas non‐histone‐mediated lactylation alters protein structure, function, and location [44]. Functionally, lactylation encourages immunosuppression and immune escape by maintaining the homeostasis of immune cells and factors. Lactylation also regulates oncogene expression. Metabolic homeostasis, cell cycle, cell death, and cancer cell plasticity are collectively regulated by lactate and lactylation. Importantly, the tumourigenic mechanisms related to lactylation are not independent of each other; instead, they exist cooperatively (Figure 2). In addition, researchers have observed that, compared to that in normal liver tissue from clinical samples, differentially lactylated proteins in liver cancer tissue and lung metastasis tissue are related to other biological processes, such as translation, ribosomal structure and biogenesis, protein turnover and chaperones, and some signalling pathways, including Notch and AMPK, which render further studies of lactylation modifications in tumour progression crucial [155].

**FIGURE 2:**
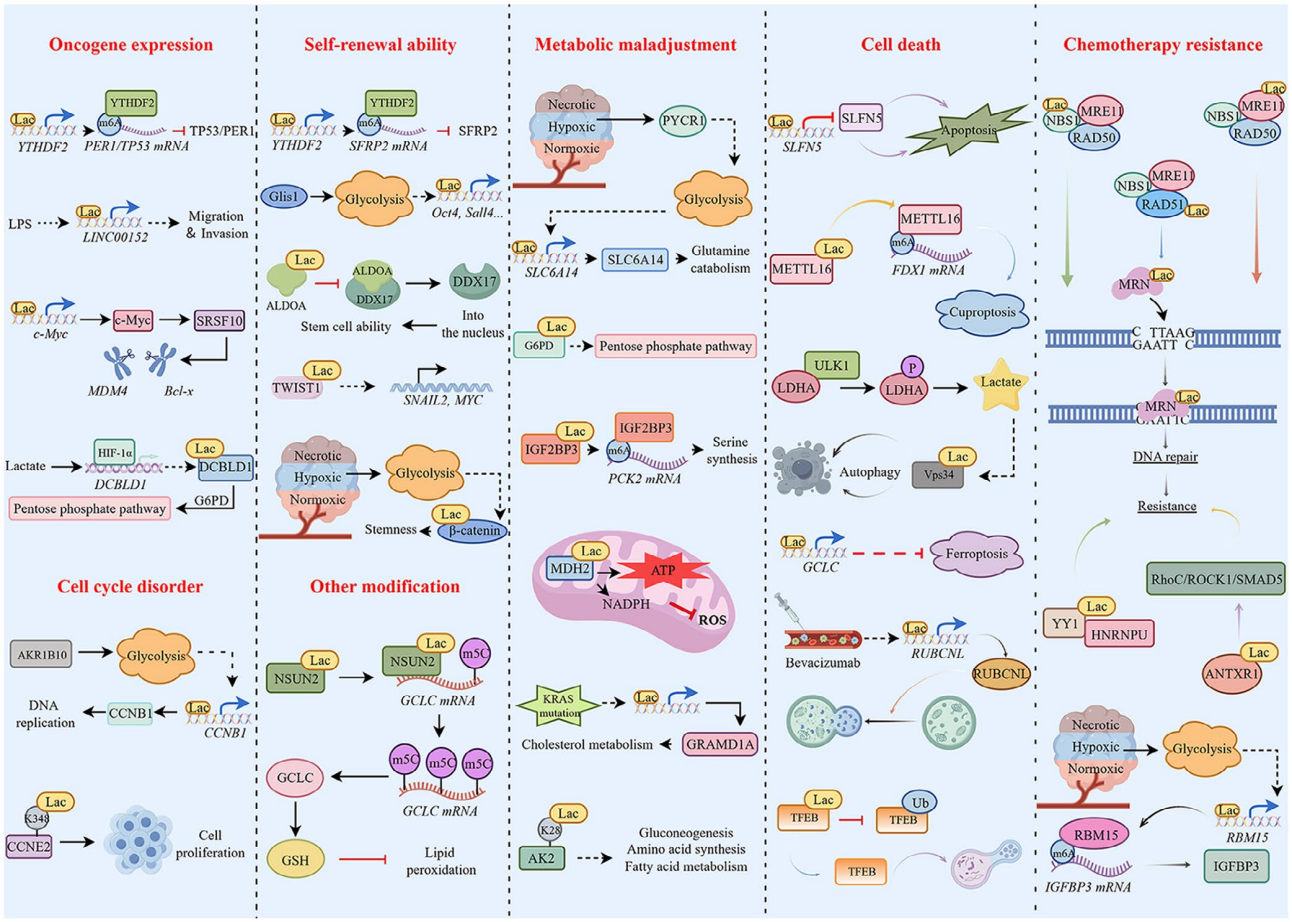
Lactylation modification promotes tumour progression. Lactylation is one of the newly discovered epigenetic modifications in the glycolytic pathway, which is regulated by multiple writers, erasers, and readers. Lactylation, in the context of glycolysis, accelerates cancer progression through various pathways such as metabolic disorders, cell death, cell pluripotency, cell cycle disruption, and treatment resistance (by Figdraw).

## 5 Lactate and Lactylation in Cancer Therapy

Lactate metabolism has shifted from being “waste in glycolysis” in the traditional sense to an important central link in tumour metabolic regulation. In recent years, lactate has been shown to not only participate in the metabolic reprogramming of tumour cells as an energy metabolite but also directly regulate protein functions through lactylation, especially histone lactylation, affecting gene transcription, immune evasion, tumour invasion and treatment tolerance [111, 161, 162]. Therefore, a tumour treatment strategy centered on “lactate–lactylation” may be beneficial and is discussed herein.

### 5.1 Inhibiting Lactate Production

The Warburg effect is key to lactate production in tumour cells, and LDHA is the key enzyme that catalyses the reversible interconversion between pyruvate and lactate. LDHA is highly expressed in various tumour cells and promotes tumour progression and drug resistance [163]. LDHA inhibitors directly reduce lactate production and hinder lactylation modifications [161]. For example, FX11 inhibits tumour cell proliferation by suppressing lactate and modulating oxidative stress in preclinical models [161, 162]. LDHA inhibition can also reduce the immunoregulation of Treg cells in the microenvironment [164]. Highly selective GNE‐140 reduces LDHA activity and increases tumour cell death in preclinical models [161, 162].

Lactate originates from glycolysis, and blocking glucose entry into glycolysis can reduce lactate [163]. GLUT1 helps glucose enter cells, and GLUT1 inhibitors, such as BAY‐876, reduce glucose uptake and decrease glycolysis and lactate production [165, 166]. The first step in glycolysis depends on hexokinase 2 (HK2) [163]. In preclinical models, the HK2 inhibitor 3‐bromopyruvate (3‐BP) reduces glycolysis, decreases lactate levels, and weakens the immune escape ability of tumour [167]. 2‐DG, a hexokinase inhibitor, has been evaluated in a Phase I trial in combination with docetaxel in patients with advanced solid tumours, where it showed acceptable tolerability, although its antitumour efficacy requires further clinical validation [168]. Shikonin inhibits the SUMOylation and dimerization of PKM2, the rate‐limiting enzyme in the final step of glycolysis. Shikonin further blocks the β‐catenin signalling pathway and interferes with the generation of stemness in preclinical findings [169].

### 5.2 Blocking Lactate Transport

Lactate is produced in large quantities within tumour cells. If they cannot be expelled, cells become acidic and die. Therefore, tumour cells form a “lactate generation and transport” adaptation system. The MCT family is the main lactate transporter family, among which MCT1 is responsible for lactate uptake, and MCT4 is mainly responsible for lactate excretion. They are highly expressed in various tumour cells [161, 162, 170]. AZD3965, an MCT1 inhibitor, disrupts lactate transport and exerts antitumour effects in preclinical models; it has also been evaluated in a Phase I clinical trial [161]. Similarly, the MCT4 inhibition increases the activity of CD8+ T cells and enhances the expression of chemokines CXCL9 and CXCL10 through the ROS/NF‐κB signalling pathway [171]. The combination of AZD3965 and the MCT4 inhibitor AZ93 significantly inhibits the proliferation of colorectal cancer cell lines [172]. CD147 regulates the stability and localization of MCT1 and MCT4 on the cell membrane; therefore, targeting CD147 may be a novel therapeutic strategy. AC‐73, a small‐molecule inhibitor targeting CD147, has further been shown to exhibit anti‐tumour activity in preclinical models [173, 174]. In preclinical models, MCT inhibition also enhances the anti‐tumour activity of anti‐PD‐1 [161, 166].

### 5.3 Interventions With Lactylation Modification Formation and Removal

The main source of lactylation modifications is lactyl‐CoA [170]. Enhanced glycolysis increases lactate levels, which enter the cell nucleus and generate lactyl‐CoA, leading to the lactylation of histones, such as H3K18la, H3K9la, and H4K12la [111, 170]. The regulation of lactylation mainly involves writers (causing lactylation formation), erasers (removing lactylation), and readers (recognising lactylation signals) [161, 163, 175]. Writer‐p300, the main lactylation transfer enzyme, uses lactyl‐CoA to catalyse histone lactylation. The p300 inhibitor, A485, reduces p300 activity and downregulates H3K18la levels, thereby inhibiting the expression of H3K18la‐related genes [175, 176]. The exogenous supplementation of β‐alanine, due to its similar structure to lactate, can further competitively inhibit AARS1‐mediated lactylation and reduce tumourigenicity in some preclinical models [176, 177]. Some HDACs have de‐lactylation capabilities, especially HDAC1–3. The HDAC inhibitors vorinostat, romidepsin, belinostat, and panobinostat have reached clinical use or clinical evaluation for specific malignancies; however, their effects on lactylation are not selective because HDAC inhibition simultaneously affects acetylation and lactylation, which may lead to adverse outcomes [173, 175, 177]. Some SIRTs also exhibit de‐lactylation enzyme activity, and the use of small‐molecule agonists can restore epigenetic balance. For example, the small‐molecule agonist SRT2104, which stabilises SIRT1, disrupts the stemness of gastric cancer cells and inhibits their growth [177].

### 5.4 Lactate–Lactylation Axis and Immunotherapeutic Interventions

Tumour cells produce a large amount of lactate, acidifying the microenvironment and resulting in decreased T cell proliferation, reduced NK cell killing ability, and macrophage polarization towards the M2 type [161, 162, 178, 179, 180]. A single metabolic inhibition is insufficient to inhibit tumour progression, whereas MCT inhibitors combined with anti‐PD‐1 treatment not only reduce lactate levels but also help T cells recover and strengthen immunotherapy [179, 180]. Similarly, p300 inhibition synergizes with anti‐PD‐L1 therapy to increase the anti‐tumour activity of T cells in prostate cancer [161]. LDHA inhibitors and anti‐PD‐1 can increase the infiltration of CD8+ T cells and inhibit Treg cells, thereby improving immune sensitivity [164, 172, 179]. LDHA inhibition and anti‐CTLA‐4 treatment also improve the anti‐tumour immune response [167]. One of the reasons for the poor efficacy of CAR‐T therapy in solid tumours is that lactate can induce the lactylation of both tumour and CAR‐T cells, leading to the exhaustion of CAR‐T cells and infiltration of Treg cells. LDHA inhibitors and CAR‐T cells working together can, on one hand, reduce the lactylation of H3K18 and transcription of immunosuppressive factors in tumour cells and, on the other hand, reduce the differentiation of Treg cells [97, 166].

In addition to immunotherapy, a combination of glycolysis inhibitors and anti‐angiogenic factors can improve the tumour vascular network and increase drug sensitivity [169]. In prostate cancer, LDHA inhibitors assist in radiotherapy by reducing intracellular lactate accumulation, regulating oxidative stress, and inhibiting DNA damage repair [168]. Overall, the therapeutic benefits of these combination strategies are currently supported mainly by preclinical studies, and further clinical investigation is required to determine their efficacy and safety in patients.

The future treatment direction is more likely to be a “lactate metabolism–lactylation–immunotherapy” combined strategy rather than a single‐target approach. However, lactylation remains in the early stages of research and understanding. The biggest challenge is to achieve the specific regulation of lactylation without affecting other widespread lysine modifications, such as acetylation. The representative compounds are listed in Table 1.

**TABLE 1:** Potential targets related to lactate and lactylation in cancer therapy.

Agent	Mechanism	Cancer type	References
Oxamate	LDHA inhibitor	TNBC, leukaemia, gastric cancer, cervical cancer, lung cancer	[72, 181]
FX11	LDHA inhibitor	TNBC, pancreatic cancer, B‐lymphoma, papillary thyroid carcinoma	[72, 181]
Galloflavin	LDHA inhibitor	TNBC and other cancer types	[181]
PSTMB	LDHA inhibitor	TNBC and other cancer types	[181]
Gossypol	LDHA inhibitor	Melanoma, leukaemia, breast cancer, cervical cancer, lung cancer, adrenal cancer	[72]
GNE‐140	LDHA inhibitor	Pancreatic cancer	[72]
NHI	LDHA/LDHB inhibitor	Pancreatic ductal adenocarcinoma, cervical cancer and mesothelioma	[72]
5‐ALA	LDH inhibitor	Glioblastoma	[182]
Lonidamine	MCT inhibitor	Neuroblastoma	[24]
BAY8002	MCT1 inhibitor	Haematopoietic cancer and some solid cancers	[183]
SR13800	MCT1 inhibitor	Neuroblastoma, lyphoma, and breast cancer	[183]
7ACC2	MCT1 inhibitor	Cervix cancer, breast cancer, pancreatic adenocarcinoma, malignant pleural effusion, and pharynx squamous cell carcinoma cells	[72, 88]
DIDS	MCT1/4 inhibitor	Colorectal cancer, lung cancer	[72]
CHC	MCT1/4 inhibitor	Glioblastoma, cervix cancer, breast cancer, colorectal cancer, prostate cancer, osteosarcoma, renal cell carcinoma	[19, 72, 184]
Phloretin	MCT1/4 inhibitor	Breast cancer, lung cancer	[72]
pCMBS	MCT1/4 inhibitor	Colorectal cancer	[72]
Quercetin	MCT1/4 inhibitor	Colorectal cancer, lung cancer, glioma	[72]
Simvastatin	MCT1/4 inhibitor	Lung cancer, breast cancer, prostate cancer, ovarian cancer, cervix cancer	[72]
Syrosingopine	MCT4 inhibitor	Liver cancer, lung cancer	[183]
AZD0095	MCT4 inhibitor	MC‐38 murine colon cancer model	[35]
AZD3965	MCT1/2 inhibitor (NCT 01791595)	Advanced solid tumours and B cell lymphomas	[19, 23]
AR‐C155858	MCT1/2 inhibitor	Breast cancer, cervix cancer, leukaemia	[15, 19, 72]
CNTO3899	Anti‐BSG monoclonal antibody	Human head and neck squamous cell carcinoma	[15]
AC‐73	Small‐molecule inhibitor of CD147	Leukaemia	[174]
3‐BP	HK‐2 inhibitor	Liver and pancreatic cancer	[167]
Honokiol	SIRT3 activator	Hepatocellular carcinoma	[64]
CCS1477	CBP/p300 inhibitor	Hematologic malignancies, advanced drug‐resistant prostate cancer	[44]
A485	p300 inhibitor	Colorectal cancer and hepatocellular carcinoma	[175]
Royal jelly acid	Reduces lactate production and inhibits H3K9 and H3K14 lactylation	Hepatocellular carcinoma	[117]
Evodiamine	Chinese herbal ingredient	Prostate cancer	[185]

Abbreviations: 3‐BP, 3‐Bromopyruvate; 5‐ALA, δ‐aminolevulinic acid; 7ACC2, 7‐(N‐benzyl‐N‐methylamino)‐2‐oxo‐2H‐chromene‐3‐carboxylic acid; BSG, BASIGIN; CHC, α‐cyano‐4‐hydroxycinnamate; DIDS, 4,4′‐diisothiocyanatostilbene‐2,2′‐disulfonic acid disodium salt hydrate; FX11, 3‐dihydroxy‐6‐methyl‐7‐(phenylmethyl)‐4‐propylnaphthalene‐1‐carboxylic acid; HK, hexokinase; NHI, N‐hydroxyindole; pCMBS, p‐chloromercuribenzene sulfonate; PSTMB, 1‐(phenylseleno)‐4‐(trifluoromethyl) benzene; TNBC, triple negative breast cancer.

## 6 Conclusions and Future Perspectives

The current understanding of lactate metabolism and lactylation modifications in cancer has been discussed herein, providing new insights into tumour progression and cancer therapy. Lactate is not only a metabolic fuel and potential signalling molecule but also a communicator between cancer cells and the cell microenvironment. The discovery of lactylation bridges lactate metabolism and epigenetics and improves our knowledge of the role of lactate in cancer (Figure 3). Targeting lactate metabolism and lactylation (Table 1) may eventually become a trend in tumour therapies.

**FIGURE 3:**
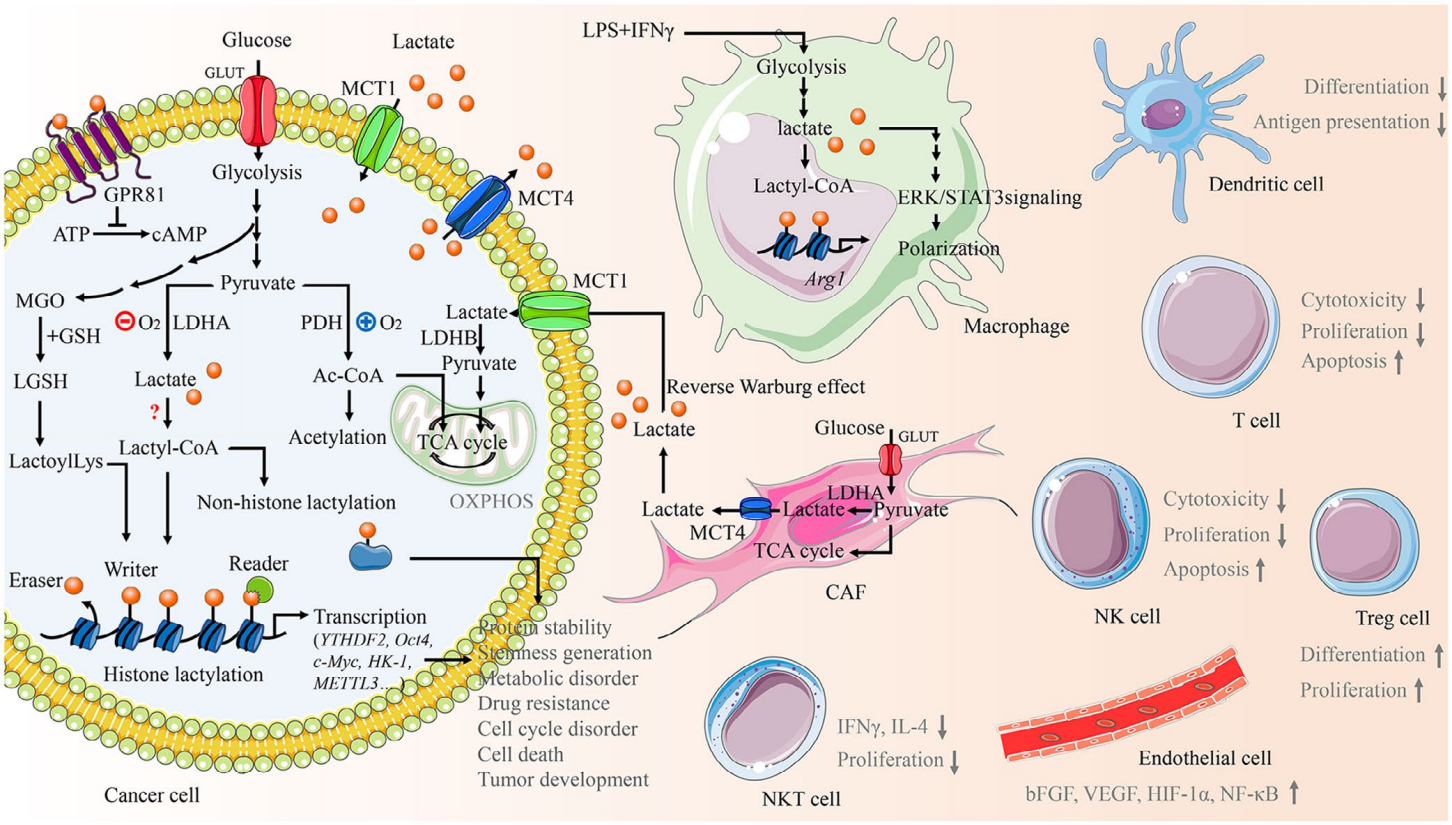
Lactate and lactylation: From formation to function in tumour progression. Lactate can not only be generated from pyruvate through the reversible reaction catalysed by LDHA but can also be transported from the extracellular space to the intracellular space via MCT1. Subsequently, lactate forms lactyl‐CoA, which dynamically modifies histones and non‐histones; this process is called lactylation modification. Lactate and lactylation in cancer cells have been shown to accelerate tumour development in various ways, including stemness generation, metabolic dysregulation, cell death, chemotherapy resistance, and cell cycle disorder. In addition, with the assistance of MCT4, lactate and lactylation promote angiogenesis and create an immunosuppressive microenvironment, which promotes tumour proliferation and metastasis (by PowerPoint). Abbreviations: LDHA, lactate dehydrogenase A; MCT1/4, monocarboxylate transporter 1/4.

Gene‐expression‐based signatures derived from lactylation‐related genes have been developed to predict prognosis, immune characteristics, and potential treatment sensitivity across multiple cancer types. In gastric cancer, a computational “lactylation score” constructed from the expression patterns of lactylation‐related genes was associated with prognosis, immune infiltration, and features of immune evasion. Patients with higher scores exhibited poorer survival and were computationally predicted to have lower responsiveness to immune checkpoint blockade. Importantly, this score represents a lactylation‐related gene‐expression signature rather than a direct measurement of cellular or protein lactylation [186]. Similarly, a lactylation‐related gene‐expression risk signature has been established in hepatocellular carcinoma. Computational drug‐sensitivity analyses predicted that patients in the high‐risk group might be more sensitive to several chemotherapeutic agents and sorafenib, whereas those in the low‐risk group showed predicted sensitivity to lapatinib and FH535 [187]. Comparable lactylation‐related gene‐expression models have also been developed in colorectal, breast, pancreatic, ovarian, prostate, and lung adenocarcinomas, revealing associations with prognosis, immune characteristics, and predicted sensitivity to immunotherapy or anticancer drugs [188, 189, 190, 191, 192, 193, 194, 195]. Immune‐associated lactylation signatures have likewise been proposed. In these models, higher lactylation‐related gene‐expression scores have been associated with reduced infiltration of CD8+ T cells, γδ T cells, NKT cells, and NK cells, suggesting a less favourable immune microenvironment [196]. Overall, these models should be interpreted as transcriptomic surrogate signatures associated with lactylation‐related biology rather than as direct measurements of lactylation, and their predicted therapeutic responses require prospective clinical validation.

Tumour cells rely heavily on glycolysis, which leads to elevated lactate levels. Lactate serves not only as an energy substrate but also as a substrate for post‐translational modifications. Histone lactylation directly influences chromatin accessibility and transcriptional status, whereas non‐histone lactylation regulates signalling pathways, homeostasis, and repair mechanisms. Lactate accumulation promotes an immunosuppressive microenvironment, and lactylation may play a role in immune cell polarization and immune evasion. Lactylation can further affect DNA damage repair, antioxidant responses, and other pathways, thereby contributing to the resistance to radiotherapy, chemotherapy, and targeted therapies, making it a potential biomarker or therapeutic target for treatment sensitivity. However, many unresolved questions and controversies remain in the field of lactate–lactylation: (1) Although candidate “writers,” “erasers,” and “readers” have been reported, the specificity, substrate profiles, and regulatory mechanisms of these enzymes are still unclear, particularly regarding the writing and selective recognition mechanisms of non‐histone lactylation, which lack definitive evidence. The competition with other post‐translational modifications, priority selection, and whether dedicated eraser enzymes exist remain inconclusive; (2) The relationship between lactylation and other post‐translational modifications such as acetylation and methylation, whether competitive or synergistic, has been poorly studied; (3) Lactylation levels and functions may vary significantly across different tumour types, stages, and microenvironments, yet no unified classification system or molecular stratification tool has been established; (4) The relative contributions of L‐lactate versus D‐lactate remain undefined; (5) How lactate/lactylation affects immune cells, such as macrophage M1/M2 polarization, T cell function, and immune checkpoint expression, is not fully understood; (6) Although lactylation correlates with changes in sensitivity to radiotherapy, chemotherapy, and immunotherapy, its direct targets and sensitization mechanisms have not been systematically validated; (7) In the tumour microenvironment, is lactate accumulation primarily affecting immune cell lactylation first, or does tumour‐intrinsic lactylation alter the tumour phenotype earlier on?

All the unknowns represent potential directions for exploration, such as (1) developing precise detection and imaging technologies for lactylation, as well as a lactylation “database” and visualization platform; (2) identifying and validating lactylation writers, erasers, and readers, systematically screening regulatory factors, constructing a complete regulatory network, and elucidating their interactions with signalling pathways; (3) building large‐scale molecular models of lactylation functions; (4) mining lactylation modification profiles as precise biomarkers for prognoses, treatment response predictions, and drug response monitoring; and (5) enhancing the efficacy of immunotherapy or targeted therapies by modulating lactate metabolism (e.g., inhibiting LDH and MCTs) and intervening in lactylation. Once these complex challenges are resolved, tumour progression may be controllable by regulating the lactate–lactylation network and subsequently extending patient survival.

## Author Contributions

Qi Zhang designed and wrote the manuscript. Zhenzhen Han revised the manuscript. Youjia Duan collected the materials. All authors read and approved the final manuscript.

## Funding

This work was supported by National Natural Science Foundation of China, 82303090.

## Conflicts of Interest

The authors declare no conflicts of interest.

## Data Availability

Data sharing not applicable to this article as no datasets were generated or analysed during the current study.

## References

[1] R. Pérez‐Tomás and I. Pérez‐Guillén , “Lactate in the Tumor Microenvironment: An Essential Molecule in Cancer Progression and Treatment,” Cancers 12, no. 11 (2020): 3244, 10.3390/cancers12113244. PMC7693872 33153193

[2] K. G. de la Cruz‐López , L. J. Castro‐Muñoz , D. O. Reyes‐Hernández , A. García‐Carrancá , and J. Manzo‐Merino , “Lactate in the Regulation of Tumor Microenvironment and Therapeutic Approaches,” Frontiers in Oncology 9 (2019): 1143, 10.3389/fonc.2019.01143. PMC6839026 31737570

[3] Y. Feng , Y. Xiong , T. Qiao , X. Li , L. Jia , and Y. Han , “Lactate Dehydrogenase A: A Key Player in Carcinogenesis and Potential Target in Cancer Therapy,” Cancer Medicine 7, no. 12 (2018): 6124–6136, 10.1002/cam4.1820. PMC6308051 30403008

[4] L. Sun , H. Zhang , and P. Gao , “Metabolic Reprogramming and Epigenetic Modifications on the Path to Cancer,” Protein & Cell 13, no. 12 (2022): 877–919, 10.1007/s13238-021-00846-7. PMC9243210 34050894

[5] A. N. Chen , Y. Luo , Y. H. Yang , et al., “Lactylation, a Novel Metabolic Reprogramming Code: Current Status and Prospects,” Frontiers in Immunology 12 (2021): 688910, 10.3389/fimmu.2021.688910. PMC8222712 34177945

[6] Y. P. Wang and Q. Y. Lei , “Metabolic Recoding of Epigenetics in Cancer,” Cancer Communications 38, no. 1 (2018): 25, 10.1186/s40880-018-0302-3. PMC5993135 29784032

[7] L. T. Izzo and K. E. Wellen , “Histone Lactylation Links Metabolism and Gene Regulation,” Nature 574, no. 7779 (2019): 492–493, 10.1038/d41586-019-03122-1. 31645737

[8] A. N. Brodsky , D. C. Odenwelder , and S. W. Harcum , “High Extracellular Lactate Causes Reductive Carboxylation in Breast Tissue Cell Lines Grown Under Normoxic Conditions,” PLoS One 14, no. 6 (2019): e0213419, 10.1371/journal.pone.0213419. PMC6557470 31181081

[9] J. Ding , J. E. Karp , and A. Emadi , “Elevated Lactate Dehydrogenase (LDH) Can Be a Marker of Immune Suppression in Cancer: Interplay Between Hematologic and Solid Neoplastic Clones and Their Microenvironments,” Cancer Biomarkers 19, no. 4 (2017): 353–363, 10.3233/cbm-160336. PMC13020749 28582845

[10] M. Ždralević , A. Brand , L. Di Ianni , et al., “Double Genetic Disruption of Lactate Dehydrogenases A and B Is Required to Ablate the ‘Warburg Effect’ Restricting Tumor Growth to Oxidative Metabolism,” Journal of Biological Chemistry 293, no. 41 (2018): 15947–15961, 10.1074/jbc.RA118.004180. PMC6187639 30158244

[11] A. P. Halestrap , “The Monocarboxylate Transporter Family—Structure and Functional Characterization,” IUBMB Life 64, no. 1 (2012): 1–9, 10.1002/iub.573. 22131303

[12] K. M. Kennedy and M. W. Dewhirst , “Tumor Metabolism of Lactate: The Influence and Therapeutic Potential for MCT and CD147 Regulation,” Future Oncology 6, no. 1 (2010): 127–148, 10.2217/fon.09.145. PMC2819205 20021214

[13] V. L. Payen , E. Mina , V. F. Van Hée , P. E. Porporato , and P. Sonveaux , “Monocarboxylate Transporters in Cancer,” Molecular Metabolism 33 (2020): 48–66, 10.1016/j.molmet.2019.07.006. PMC7056923 31395464

[14] F. Baltazar , J. Afonso , M. Costa , and S. Granja , “Lactate Beyond a Waste Metabolite: Metabolic Affairs and Signaling in Malignancy,” Frontiers in Oncology 10 (2020): 231, 10.3389/fonc.2020.00231. PMC7093491 32257942

[15] I. Marchiq and J. Pouysségur , “Hypoxia, Cancer Metabolism and the Therapeutic Benefit of Targeting Lactate/H(+) Symporters,” Journal of Molecular Medicine (Berlin, Germany) 94, no. 2 (2016): 155–171, 10.1007/s00109-015-1307-x. PMC4762928 26099350

[16] E. J. Choi , Y. Y. Jang , E. J. Choi , and C. J. Oh , “The Role of Lactate in Immune Regulation: A Metabolic Rheostat via Transporters, Receptors, and Epigenetic Modifiers,” Cells 14, no. 14 (2025): 1096, 10.3390/cells14141096. PMC12294089 40710349

[17] P. Stepka , V. Vsiansky , M. Raudenska , J. Gumulec , V. Adam , and M. Masarik , “Metabolic and Amino Acid Alterations of the Tumor Microenvironment,” Current Medicinal Chemistry 28, no. 7 (2021): 1270–1289, 10.2174/0929867327666200207114658. 32031065

[18] L. Hou , Y. Zhao , G. Q. Song , et al., “Interfering Cellular Lactate Homeostasis Overcomes Taxol Resistance of Breast Cancer Cells Through the microRNA‐124‐Mediated Lactate Transporter (MCT1) Inhibition,” Cancer Cell International 19 (2019): 193, 10.1186/s12935-019-0904-0. PMC6657142 31367191

[19] J. R. Doherty and J. L. Cleveland , “Targeting Lactate Metabolism for Cancer Therapeutics,” Journal of Clinical Investigation 123, no. 9 (2013): 3685–3692, 10.1172/jci69741. PMC3754272 23999443

[20] P. Vaupel and G. Multhoff , “Hypoxia‐/HIF‐1α‐Driven Factors of the Tumor Microenvironment Impeding Antitumour Immune Responses and Promoting Malignant Progression,” Advances in Experimental Medicine and Biology 1072 (2018): 171–175, 10.1007/978-3-319-91287-5_27. 30178341

[21] D. Mishra and D. Banerjee , “Metabolic Interactions Between Tumor and Stromal Cells in the Tumor Microenvironment,” Advances in Experimental Medicine and Biology 1350 (2021): 101–121, 10.1007/978-3-030-83282-7_5. 34888846

[22] J. Yang , B. Ren , G. Yang , et al., “The Enhancement of Glycolysis Regulates Pancreatic Cancer Metastasis,” Cellular and Molecular Life Sciences 77, no. 2 (2020): 305–321, 10.1007/s00018-019-03278-z. PMC11104916 31432232

[23] K. Renner , K. Singer , G. E. Koehl , et al., “Metabolic Hallmarks of Tumor and Immune Cells in the Tumor Microenvironment,” Frontiers in Immunology 8 (2017): 248, 10.3389/fimmu.2017.00248. PMC5340776 28337200

[24] S. Romero‐Garcia , M. M. Moreno‐Altamirano , H. Prado‐Garcia , and F. J. Sánchez‐García , “Lactate Contribution to the Tumor Microenvironment: Mechanisms, Effects on Immune Cells and Therapeutic Relevance,” Frontiers in Immunology 7 (2016): 52, 10.3389/fimmu.2016.00052. PMC4754406 26909082

[25] I. Terrén , A. Orrantia , J. Vitallé , O. Zenarruzabeitia , and F. Borrego , “NK Cell Metabolism and Tumor Microenvironment,” Frontiers in Immunology 10 (2019): 2278, 10.3389/fimmu.2019.02278. PMC6769035 31616440

[26] J. Pérez‐Escuredo , V. F. Van Hée , M. Sboarina , et al., “Monocarboxylate Transporters in the Brain and in Cancer,” Biochimica et Biophysica Acta 1863, no. 10 (2016): 2481–2497, 10.1016/j.bbamcr.2016.03.013. PMC4990061 26993058

[27] H. Deng , A. Kan , N. Lyu , et al., “Tumor‐Derived Lactate Inhibit the Efficacy of Lenvatinib Through Regulating PD‐L1 Expression on Neutrophil in Hepatocellular Carcinoma,” Journal for Immunotherapy of Cancer 9, no. 6 (2021): e002305, 10.1136/jitc-2020-002305. PMC8231064 34168004

[28] X. Mu , W. Shi , Y. Xu , et al., “Tumor‐Derived Lactate Induces M2 Macrophage Polarization via the Activation of the ERK/STAT3 Signaling Pathway in Breast Cancer,” Cell Cycle 17, no. 4 (2018): 428–438, 10.1080/15384101.2018.1444305. PMC5927648 29468929

[29] A. Zhang , Y. Xu , H. Xu , et al., “Lactate‐Induced M2 Polarization of Tumor‐Associated Macrophages Promotes the Invasion of Pituitary Adenoma by Secreting CCL17,” Theranostics 11, no. 8 (2021): 3839–3852, 10.7150/thno.53749. PMC7914368 33664865

[30] L. F. Somarribas Patterson and S. A. Vardhana , “Metabolic Regulation of the Cancer‐Immunity Cycle,” Trends in Immunology 42, no. 11 (2021): 975–993, 10.1016/j.it.2021.09.002. PMC8556351 34610889

[31] N. Liu , J. Luo , D. Kuang , et al., “Lactate Inhibits ATP6V0d2 Expression in Tumor‐Associated Macrophages to Promote HIF‐2α‐Mediated Tumor Progression,” Journal of Clinical Investigation 129, no. 2 (2019): 631–646, 10.1172/jci123027. PMC6355226 30431439

[32] S. Cassim and J. Pouyssegur , “Tumor Microenvironment: A Metabolic Player That Shapes the Immune Response,” International Journal of Molecular Sciences 21, no. 1 (2019): 157, 10.3390/ijms21010157. PMC6982275 31881671

[33] D. J. Samuvel , K. P. Sundararaj , A. Nareika , M. F. Lopes‐Virella , and Y. Huang , “Lactate Boosts TLR4 Signaling and NF‐kappaB Pathway‐Mediated Gene Transcription in Macrophages via Monocarboxylate Transporters and MD‐2 Up‐Regulation,” Journal of Immunology 182, no. 4 (2009): 2476–2484, 10.4049/jimmunol.0802059. PMC2673542 19201903

[34] T. P. Brown and V. Ganapathy , “Lactate/GPR81 Signaling and Proton Motive Force in Cancer: Role in Angiogenesis, Immune Escape, Nutrition, and Warburg Phenomenon,” Pharmacology & Therapeutics 206 (2020): 107451, 10.1016/j.pharmthera.2019.107451. 31836453

[35] C. Harmon , C. O'Farrelly , and M. W. Robinson , “The Immune Consequences of Lactate in the Tumor Microenvironment,” Advances in Experimental Medicine and Biology 1259 (2020): 113–124, 10.1007/978-3-030-43093-1_7. 32578174

[36] I. San‐Millán and G. A. Brooks , “Reexamining Cancer Metabolism: Lactate Production for Carcinogenesis Could Be the Purpose and Explanation of the Warburg Effect,” Carcinogenesis 38, no. 2 (2017): 119–133, 10.1093/carcin/bgw127. PMC5862360 27993896

[37] I. Nenu , G. A. Gafencu , T. Popescu , and G. Kacso , “Lactate—A New Frontier in the Immunology and Therapy of Prostate Cancer,” Journal of Cancer Research and Therapeutics 13, no. 3 (2017): 406–411, 10.4103/0973-1482.163692. 28862200

[38] U. E. Martinez‐Outschoorn , M. Prisco , A. Ertel , et al., “Ketones and Lactate Increase Cancer Cell ‘Stemness’, Driving Recurrence, Metastasis and Poor Clinical Outcome in Breast Cancer: Achieving Personalized Medicine via Metabolo‐Genomics,” Cell Cycle 10, no. 8 (2011): 1271–1286, 10.4161/cc.10.8.15330. PMC3117136 21512313

[39] T. Wang , S. Shigdar , M. P. Gantier , et al., “Cancer Stem Cell Targeted Therapy: Progress Amid Controversies,” Oncotarget 6, no. 42 (2015): 44191–44206, 10.18632/oncotarget.6176. PMC4792551 26496035

[40] M. A. Khajah , S. Khushaish , and Y. A. Luqmani , “Lactate Dehydrogenase A or B Knockdown Reduces Lactate Production and Inhibits Breast Cancer Cell Motility In Vitro,” Frontiers in Pharmacology 12 (2021): 747001, 10.3389/fphar.2021.747001. PMC8564068 34744727

[41] D. Zhang , Z. Tang , H. Huang , et al., “Metabolic Regulation of Gene Expression by Histone Lactylation,” Nature 574, no. 7779 (2019): 575–580, 10.1038/s41586-019-1678-1. PMC6818755 31645732

[42] E. L. Varner , S. Trefely , D. Bartee , et al., “Quantification of Lactoyl‐CoA (Lactyl‐CoA) by Liquid Chromatography Mass Spectrometry in Mammalian Cells and Tissues,” Open Biology 10, no. 9 (2020): 200187, 10.1098/rsob.200187. PMC7536085 32961073

[43] X. Dai , X. Lv , E. W. Thompson , and K. K. Ostrikov , “Histone Lactylation: Epigenetic Mark of Glycolytic Switch,” Trends in Genetics 38, no. 2 (2022): 124–127, 10.1016/j.tig.2021.09.009. 34627643

[44] J. H. Wang , L. Mao , J. Wang , et al., “Beyond Metabolic Waste: Lysine Lactylation and Its Potential Roles in Cancer Progression and Cell Fate Determination,” Cellular Oncology (Dordrecht) 46, no. 3 (2023): 465–480, 10.1007/s13402-023-00775-z. PMC12974629 36656507

[45] H. Li , L. Sun , P. Gao , and H. Hu , “Lactylation in Cancer: Current Understanding and Challenges,” Cancer Cell 42, no. 11 (2024): 1803–1807, 10.1016/j.ccell.2024.09.006. 39393355

[46] D. O. Gaffney , E. Q. Jennings , C. C. Anderson , et al., “Non‐Enzymatic Lysine Lactoylation of Glycolytic Enzymes,” Cell Chemical Biology 27, no. 2 (2020): 206–213, 10.1016/j.chembiol.2019.11.005. PMC7395678 31767537

[47] L. Li , K. Chen , T. Wang , et al., “Glis1 Facilitates Induction of Pluripotency via an Epigenome‐Metabolome‐Epigenome Signalling Cascade,” Nature Metabolism 2, no. 9 (2020): 882–892, 10.1038/s42255-020-0267-9. 32839595

[48] C. Zhang , S. Huang , H. Zhuang , et al., “YTHDF2 Promotes the Liver Cancer Stem Cell Phenotype and Cancer Metastasis by Regulating OCT4 Expression via m6A RNA Methylation,” Oncogene 39, no. 23 (2020): 4507–4518, 10.1038/s41388-020-1303-7. 32366907

[49] H. Chen , Y. Li , H. Li , et al., “NBS1 Lactylation Is Required for Efficient DNA Repair and Chemotherapy Resistance,” Nature 631, no. 8021 (2024): 663–669, 10.1038/s41586-024-07620-9. PMC11254748 38961290

[50] Z. Zong , F. Xie , S. Wang , et al., “Alanyl‐tRNA Synthetase, AARS1, Is a Lactate Sensor and Lactyltransferase That Lactylates p53 and Contributes to Tumorigenesis,” Cell 187, no. 10 (2024): 2375–2392, 10.1016/j.cell.2024.04.002. 38653238

[51] J. Ju , H. Zhang , M. Lin , et al., “The Alanyl‐tRNA Synthetase AARS1 Moonlights as a Lactyltransferase to Promote YAP Signaling in Gastric Cancer,” Journal of Clinical Investigation 134, no. 10 (2024): e174587, 10.1172/jci174587. PMC11093599 38512451

[52] C. Sun , X. Li , Q. Teng , et al., “Targeting Platinum‐Resistant Ovarian Cancer by Disrupting Histone and RAD51 Lactylation,” Theranostics 15, no. 7 (2025): 3055–3075, 10.7150/thno.104858. PMC11898288 40083924

[53] L. Sun , Y. Zhang , B. Yang , et al., “Lactylation of METTL16 Promotes Cuproptosis via m(6)A‐Modification on FDX1 mRNA in Gastric Cancer,” Nature Communications 14, no. 1 (2023): 6523, 10.1038/s41467-023-42025-8. PMC10589265 37863889

[54] J. He , T. Lai , Y. Zhao , et al., “HAT1 Functions as a Lactyltransferase and Mediates RPA1 Lactylation to Promote DNA Repair and Radioresistance in Lung Adenocarcinoma,” Cell Death & Disease 16, no. 1 (2025): 851, 10.1038/s41419-025-08113-x. PMC12639135 41271679

[55] B. Xie , M. Zhang , J. Li , et al., “KAT8‐Catalyzed Lactylation Promotes eEF1A2‐Mediated Protein Synthesis and Colorectal Carcinogenesis,” Proceedings of the National Academy of Sciences of the United States of America 121, no. 8 (2024): e2314128121, 10.1073/pnas.2314128121. PMC10895275 38359291

[56] Y. Sun , H. Wang , Z. Cui , et al., “Lactylation in Cancer Progression and Drug Resistance,” Drug Resistance Updates 81 (2025): 101248, 10.1016/j.drup.2025.101248. 40287994

[57] Y. Tang , C. Dai , H. Yang , et al., “KAT8‐Mediated MDH2 Lactylation Promotes Renal Cancer Progression by Enhancing Mitochondrial Function and Stress Resistance,” International Journal of Biological Macromolecules 322, no. Pt 1 (2025): 146571, 10.1016/j.ijbiomac.2025.146571. 40769364

[58] C. Moreno‐Yruela , D. Zhang , W. Wei , et al., “Class I Histone Deacetylases (HDAC1‐3) Are Histone Lysine Delactylases,” Science Advances 8, no. 3 (2022): eabi6696, 10.1126/sciadv.abi6696. PMC8769552 35044827

[59] F. Wang , C. B. Marshall , and M. Ikura , “Transcriptional/Epigenetic Regulator CBP/p300 in Tumorigenesis: Structural and Functional Versatility in Target Recognition,” Cellular and Molecular Life Sciences 70, no. 21 (2013): 3989–4008, 10.1007/s00018-012-1254-4. PMC11113169 23307074

[60] J. Zhong , L. Ding , L. R. Bohrer , et al., “p300 Acetyltransferase Regulates Androgen Receptor Degradation and PTEN‐Deficient Prostate Tumorigenesis,” Cancer Research 74, no. 6 (2014): 1870–1880, 10.1158/0008-5472.Can-13-2485. PMC3971883 24480624

[61] A. C. West and R. W. Johnstone , “New and Emerging HDAC Inhibitors for Cancer Treatment,” Journal of Clinical Investigation 124, no. 1 (2014): 30–39, 10.1172/jci69738. PMC3871231 24382387

[62] R. Jenke , N. Reßing , F. K. Hansen , A. Aigner , and T. Büch , “Anticancer Therapy With HDAC Inhibitors: Mechanism‐Based Combination Strategies and Future Perspectives,” Cancers 13, no. 4 (2021): 634, 10.3390/cancers13040634. PMC7915831 33562653

[63] D. Cai , X. Yuan , D. Q. Cai , et al., “Integrative Analysis of Lactylation‐Related Genes and Establishment of a Novel Prognostic Signature for Hepatocellular Carcinoma,” Journal of Cancer Research and Clinical Oncology 149 (2023): 11517–11530, 10.1007/s00432-023-04947-0. PMC11796770 37400571

[64] J. Jin , L. Bai , D. Wang , et al., “SIRT3‐Dependent Delactylation of Cyclin E2 Prevents Hepatocellular Carcinoma Growth,” EMBO Reports 24, no. 5 (2023): e56052, 10.15252/embr.202256052. PMC10157311 36896611

[65] C. Chen , Y. Zhang , Y. Zang , et al., “SIRT3 Functions as an Eraser of Histone H3K9 Lactylation to Modulate Transcription for Inhibiting the Progression of Esophageal Cancer,” Molecular & Cellular Proteomics 24, no. 5 (2025): 100973, 10.1016/j.mcpro.2025.100973. PMC12144510 40252727

[66] X. Gao , C. Pang , Z. Fan , Y. Wang , Y. Duan , and H. Zhan , “Regulation of Newly Identified Lysine Lactylation in Cancer,” Cancer Letters 587 (2024): 216680, 10.1016/j.canlet.2024.216680. 38346584

[67] S. Tsukihara , Y. Akiyama , S. Shimada , et al., “Delactylase Effects of SIRT1 on a Positive Feedback Loop Involving the H19‐Glycolysis‐Histone Lactylation in Gastric Cancer,” Oncogene 44, no. 11 (2025): 724–738, 10.1038/s41388-024-03243-6. 39658647

[68] G. A. Nickel , N. J. Pederson , Faheem , Z. Yang , J. Bulf , and K. L. Diehl , “Sirtuin 6 Is a Histone Delactylase,” Journal of Biological Chemistry 301, no. 12 (2025): 110795, 10.1016/j.jbc.2025.110795. PMC12664031 41062064

[69] G. Zhai , Z. Niu , Z. Jiang , et al., “DPF2 Reads Histone Lactylation to Drive Transcription and Tumorigenesis,” Proceedings of the National Academy of Sciences of the United States of America 121, no. 50 (2024): e2421496121, 10.1073/pnas.2421496121. PMC11648877 39636855

[70] X. Peng and J. Du , “Histone and Non‐Histone Lactylation: Molecular Mechanisms, Biological Functions, Diseases, and Therapeutic Targets,” Molecular Biomedicine 6, no. 1 (2025): 38, 10.1186/s43556-025-00275-6. PMC12146230 40484921

[71] Y. Qiao , Y. Liu , R. Ran , et al., “Lactate Metabolism and Lactylation in Breast Cancer: Mechanisms and Implications,” Cancer Metastasis Reviews 44, no. 2 (2025): 48, 10.1007/s10555-025-10264-4. PMC12037681 40295451

[72] Z. H. Wang , W. B. Peng , P. Zhang , X. P. Yang , and Q. Zhou , “Lactate in the Tumour Microenvironment: From Immune Modulation to Therapy,” eBioMedicine 73 (2021): 103627, 10.1016/j.ebiom.2021.103627. PMC8524104 34656878

[73] S. Dichtl , L. Lindenthal , L. Zeitler , et al., “Lactate and IL6 Define Separable Paths of Inflammatory Metabolic Adaptation,” Science Advances 7, no. 26 (2021): eabg3505, 10.1126/sciadv.abg3505. PMC8221612 34162546

[74] M. V. Liberti and J. W. Locasale , “Histone Lactylation: A New Role for Glucose Metabolism,” Trends in Biochemical Sciences 45, no. 3 (2020): 179–182, 10.1016/j.tibs.2019.12.004. 31901298

[75] F. Guo , M. Hu , D. Huang , et al., “Human Regulatory Macrophages Are Potent in Suppression of the Xenoimmune Response via Indoleamine‐2,3‐Dioxygenase‐Involved Mechanism(s),” Xenotransplantation 24, no. 5 (2017): e12326, 10.1111/xen.12326. 28771838

[76] C. Huang , L. Xue , X. Lin , Y. Shen , and X. Wang , “Histone Lactylation‐Driven GPD2 Mediates M2 Macrophage Polarization to Promote Malignant Transformation of Cervical Cancer Progression,” DNA and Cell Biology 43, no. 12 (2024): 605–618, 10.1089/dna.2024.0122. 39504115

[77] T. Dang , Y. You , L. Wei , et al., “ICAT Drives Lactylation of Tumor‐Associated Macrophages via the c‐Myc‐ENO1 Axis to Promote Cervical Cancer Progression,” Free Radical Biology & Medicine 241 (2025): 316–329, 10.1016/j.freeradbiomed.2025.09.031. 40976412

[78] M. Li , P. Sun , B. Tu , G. Deng , D. Li , and W. He , “Hypoxia Conduces the Glioma Progression by Inducing M2 Macrophage Polarization via Elevating TNFSF9 Level in a Histone‐Lactylation‐Dependent Manner,” American Journal of Physiology. Cell Physiology 327, no. 2 (2024): C487–c504, 10.1152/ajpcell.00124.2024. 39010835

[79] X. Deng , Y. Huang , J. Zhang , et al., “Histone Lactylation Regulates PRKN‐Mediated Mitophagy to Promote M2 Macrophage Polarization in Bladder Cancer,” International Immunopharmacology 148 (2025): 114119, 10.1016/j.intimp.2025.114119. 39854875

[80] A. De Leo , A. Ugolini , X. Yu , et al., “Glucose‐Driven Histone Lactylation Promotes the Immunosuppressive Activity of Monocyte‐Derived Macrophages in Glioblastoma,” Immunity 57, no. 5 (2024): 1105–1123, 10.1016/j.immuni.2024.04.006. PMC11114377 38703775

[81] Y. Zhao , J. Jiang , P. Zhou , et al., “H3K18 Lactylation‐Mediated VCAM1 Expression Promotes Gastric Cancer Progression and Metastasis via AKT‐mTOR‐CXCL1 Axis,” Biochemical Pharmacology 222 (2024): 116120, 10.1016/j.bcp.2024.116120. 38461905

[82] J. Yang , X. Yu , M. Xiao , et al., “Histone Lactylation‐Driven Feedback Loop Modulates Cholesterol‐Linked Immunosuppression in Pancreatic Cancer,” Gut 74, no. 11 (2025): 1859–1872, 10.1136/gutjnl-2024-334361. PMC12573332 40467104

[83] J. Gu , J. Zhou , Q. Chen , et al., “Tumor Metabolite Lactate Promotes Tumorigenesis by Modulating MOESIN Lactylation and Enhancing TGF‐Beta Signaling in Regulatory T Cells,” Cell Reports 39, no. 12 (2022): 110986, 10.1016/j.celrep.2022.110986. 35732125

[84] T. Wang , Z. Ye , Z. Li , et al., “Lactate‐Induced Protein Lactylation: A Bridge Between Epigenetics and Metabolic Reprogramming in Cancer,” Cell Proliferation 56 (2023): e13478, 10.1111/cpr.13478. PMC10542650 37060186

[85] Q. Xue , W. Peng , S. Zhang , et al., “Lactylation‐Driven TNFR2 Expression in Regulatory T Cells Promotes the Progression of Malignant Pleural Effusion,” Journal for Immunotherapy of Cancer 12, no. 12 (2024): e010040, 10.1136/jitc-2024-010040. PMC11683941 39721754

[86] L. Ye , X. Xiang , Z. Wang , et al., “H3K18 Lactylation in Cancer‐Associated Fibroblasts Drives Malignant Pleural Effusion Progression via TNFR2(+) T(Reg) Recruitment,” Experimental & Molecular Medicine 57, no. 11 (2025): 2671–2685, 10.1038/s12276-025-01557-3. PMC12686498 41310104

[87] X. Li , J. Peng , Y. Pang , et al., “Identification of a FOXP3(+)CD3(+)CD56(+) Population With Immunosuppressive Function in Cancer Tissues of Human Hepatocellular Carcinoma,” Scientific Reports 5 (2015): 14757, 10.1038/srep14757. PMC4594002 26437631

[88] Z. H. Wang , P. Zhang , W. B. Peng , et al., “Altered Phenotypic and Metabolic Characteristics of FOXP3(+)CD3(+)CD56(+) Natural Killer T (NKT)‐Like Cells in Human Malignant Pleural Effusion,” Oncoimmunology 12, no. 1 (2023): 2160558, 10.1080/2162402x.2022.2160558. PMC9788685 36567801

[89] L. Liang , Y. Zong , J. Huang , et al., “Lactylation Stabilizes PD‐L1 to Promote Tumor Immune Evasion and Cell Growth,” Cell Death & Disease 17, no. 1 (2026): 1, 10.1038/s41419-026-08589-1. PMC13039446 41864972

[90] Y. Zhang , H. Song , M. Li , and P. Lu , “Histone Lactylation Bridges Metabolic Reprogramming and Epigenetic Rewiring in Driving Carcinogenesis: Oncometabolite Fuels Oncogenic Transcription,” Clinical and Translational Medicine 14, no. 3 (2024): e1614, 10.1002/ctm2.1614. PMC10921234 38456209

[91] Z. W. Huang , X. N. Zhang , L. Zhang , et al., “STAT5 Promotes PD‐L1 Expression by Facilitating Histone Lactylation to Drive Immunosuppression in Acute Myeloid Leukemia,” Signal Transduction and Targeted Therapy 8, no. 1 (2023): 391, 10.1038/s41392-023-01605-2. PMC10542808 37777506

[92] C. Zhang , L. Zhou , M. Zhang , et al., “H3K18 Lactylation Potentiates Immune Escape of Non‐Small Cell Lung Cancer,” Cancer Research 84, no. 21 (2024): 3589–3601, 10.1158/0008-5472.Can-23-3513. 39137401

[93] X. Hu , W. Ouyang , H. Chen , Z. Liu , Z. Lai , and H. Yao , “Claudin‐9 (CLDN9) Promotes Gastric Cancer Progression by Enhancing the Glycolysis Pathway and Facilitating PD‐L1 Lactylation to Suppress CD8(+) T Cell Anti‐Tumor Immunity,” Cancer Pathogenesis and Therapy 3, no. 3 (2025): 253–266, 10.1016/j.cpt.2024.09.006. PMC12126740 40458308

[94] R. Wang , C. Li , Z. Cheng , et al., “H3K9 Lactylation in Malignant Cells Facilitates CD8(+) T Cell Dysfunction and Poor Immunotherapy Response,” Cell Reports 43, no. 9 (2024): 114686, 10.1016/j.celrep.2024.114686. 39216002

[95] J. Xiong , J. He , J. Zhu , et al., “Lactylation‐Driven METTL3‐Mediated RNA m(6)A Modification Promotes Immunosuppression of Tumor‐Infiltrating Myeloid Cells,” Molecular Cell 82, no. 9 (2022): 1660–1677, 10.1016/j.molcel.2022.02.033. 35320754

[96] J. Zha , J. Zhang , J. Lu , et al., “A Review of Lactate‐Lactylation in Malignancy: Its Potential in Immunotherapy,” Frontiers in Immunology 15 (2024): 1384948, 10.3389/fimmu.2024.1384948. PMC11109376 38779665

[97] T. Sun , B. Liu , Y. Li , et al., “Oxamate Enhances the Efficacy of CAR‐T Therapy Against Glioblastoma via Suppressing Ectonucleotidases and CCR8 Lactylation,” Journal of Experimental & Clinical Cancer Research 42, no. 1 (2023): 253, 10.1186/s13046-023-02815-w. PMC10540361 37770937

[98] M. Zhao , Y. Qian , L. He , et al., “Lactate‐Mediated Histone Lactylation Promotes Melanoma Angiogenesis via IL‐33/ST2 Axis,” Cell Death & Disease 16, no. 1 (2025): 701, 10.1038/s41419-025-08023-y. PMC12501017 41053006

[99] Y. Luo , Z. Yang , Y. Yu , and P. Zhang , “HIF1α Lactylation Enhances KIAA1199 Transcription to Promote Angiogenesis and Vasculogenic Mimicry in Prostate Cancer,” International Journal of Biological Macromolecules 222 (2022): 2225–2243, 10.1016/j.ijbiomac.2022.10.014. 36209908

[100] J. Yu , P. Chai , M. Xie , et al., “Histone Lactylation Drives Oncogenesis by Facilitating m(6)A Reader Protein YTHDF2 Expression in Ocular Melanoma,” Genome Biology 22, no. 1 (2021): 85, 10.1186/s13059-021-02308-z. PMC7962360 33726814

[101] Y. Li , H. Sheng , F. Ma , et al., “RNA m(6)A Reader YTHDF2 Facilitates Lung Adenocarcinoma Cell Proliferation and Metastasis by Targeting the AXIN1/Wnt/β‐Catenin Signaling,” Cell Death & Disease 12, no. 5 (2021): 479, 10.1038/s41419-021-03763-z. PMC8116339 33980824

[102] S. Zhao , J. Cheng , G. Zhou , et al., “Histone Lactylation‐Driven YTHDF2 Promotes Non‐Small Cell Lung Cancer Cell Glycolysis and Stemness by Recognizing m6A Modification of SFRP2,” Biochemical Pharmacology 240 (2025): 117097, 10.1016/j.bcp.2025.117097. 40619016

[103] J. Wang , Z. Liu , Y. Xu , et al., “Enterobacterial LPS‐Inducible LINC00152 Is Regulated by Histone Lactylation and Promotes Cancer Cells Invasion and Migration,” Frontiers in Cellular and Infection Microbiology 12 (2022): 913815, 10.3389/fcimb.2022.913815. PMC9359126 35959377

[104] M. R. Pandkar , S. Sinha , A. Samaiya , and S. Shukla , “Oncometabolite Lactate Enhances Breast Cancer Progression by Orchestrating Histone Lactylation‐Dependent c‐Myc Expression,” Translational Oncology 37 (2023): 101758, 10.1016/j.tranon.2023.101758. PMC10425713 37572497

[105] Q. Meng , H. Sun , Y. Zhang , et al., “Lactylation Stabilizes DCBLD1 Activating the Pentose Phosphate Pathway to Promote Cervical Cancer Progression,” Journal of Experimental & Clinical Cancer Research 43, no. 1 (2024): 36, 10.1186/s13046-024-02943-x. PMC10829273 38291438

[106] Z. Yang , C. Yan , J. Ma , et al., “Lactylome Analysis Suggests Lactylation‐Dependent Mechanisms of Metabolic Adaptation in Hepatocellular Carcinoma,” Nature Metabolism 5, no. 1 (2023): 61–79, 10.1038/s42255-022-00710-w. 36593272

[107] J. Jiang , D. Huang , Y. Jiang , et al., “Lactate Modulates Cellular Metabolism Through Histone Lactylation‐Mediated Gene Expression in Non‐Small Cell Lung Cancer,” Frontiers in Oncology 11 (2021): 647559, 10.3389/fonc.2021.647559. PMC8208031 34150616

[108] X. Li , Y. Yang , B. Zhang , et al., “Lactate Metabolism in Human Health and Disease,” Signal Transduction and Targeted Therapy 7, no. 1 (2022): 305, 10.1038/s41392-022-01151-3. PMC9434547 36050306

[109] Y. Zhang , Y. Wu , A. Cheng , F. Cui , Z. Yang , and J. Guo , “The Lactylation of Glucose‐6‐Phosphate Dehydrogenase Promotes Malignant Phenotypes in Cancer Cell Lines,” Molecular Biology Reports 52, no. 1 (2025): 861, 10.1007/s11033-025-10960-y. 40900394

[110] Y. Lu , J. Zhu , Y. Zhang , et al., “Lactylation‐Driven IGF2BP3‐Mediated Serine Metabolism Reprogramming and RNA m6A‐Modification Promotes Lenvatinib Resistance in HCC,” Advanced Science 11, no. 46 (2024): e2401399, 10.1002/advs.202401399. PMC11633555 39450426

[111] M. Lv , Y. Huang , Y. Chen , and K. Ding , “Lactylation Modification in Cancer: Mechanisms, Functions, and Therapeutic Strategies,” Experimental Hematology & Oncology 14, no. 1 (2025): 32, 10.1186/s40164-025-00622-x. PMC11889934 40057816

[112] Z. Li , Q. Jiang , Q. Yang , Y. Zhou , and J. Wang , “Hypoxia‐Induced PYCR1 Regulates Glycolysis and Histone Lactylation to Promote Bladder Cancer Progression and Metastasis via SLC6A14/Glutamine Metabolism,” Cancer Biology & Therapy 26, no. 1 (2025): 2546219, 10.1080/15384047.2025.2546219. PMC12355718 40808274

[113] C. Zhang , R. Yu , S. Li , et al., “KRAS Mutation Increases Histone H3 Lysine 9 Lactylation (H3K9la) to Promote Colorectal Cancer Progression by Facilitating Cholesterol Transporter GRAMD1A Expression,” Cell Death and Differentiation 32, no. 12 (2025): 2225–2238, 10.1038/s41418-025-01533-4. PMC12669710 40707783

[114] R. P. Chen , C. Y. Liu , H. L. Shao , W. W. Zheng , J. X. Wang , and X. F. Zhao , “Adenylate Kinase 2 (AK2) Promotes Cell Proliferation in Insect Development,” BMC Molecular Biology 13 (2012): 31, 10.1186/1471-2199-13-31. PMC3583204 23020757

[115] J. Liu , Y. Peng , L. Shi , et al., “Skp2 Dictates Cell Cycle‐Dependent Metabolic Oscillation Between Glycolysis and TCA Cycle,” Cell Research 31, no. 1 (2021): 80–93, 10.1038/s41422-020-0372-z. PMC7852548 32669607

[116] J. Lin , G. Liu , L. Chen , H. F. Kwok , and Y. Lin , “Targeting Lactate‐Related Cell Cycle Activities for Cancer Therapy,” Seminars in Cancer Biology 86, no. Pt 3 (2022): 1231–1243, 10.1016/j.semcancer.2022.10.009. 36328311

[117] H. Xu , L. Li , S. Wang , et al., “Royal Jelly Acid Suppresses Hepatocellular Carcinoma Tumourigenicity by Inhibiting H3 Histone Lactylation at H3K9la and H3K14la Sites,” Phytomedicine 118 (2023): 154940, 10.1016/j.phymed.2023.154940. 37453194

[118] W. Duan , W. Liu , S. Xia , et al., “Warburg Effect Enhanced by AKR1B10 Promotes Acquired Resistance to Pemetrexed in Lung Cancer‐Derived Brain Metastasis,” Journal of Translational Medicine 21, no. 1 (2023): 547, 10.1186/s12967-023-04403-0. PMC10428599 37587486

[119] T. Huang , X. Song , D. Xu , et al., “Stem Cell Programs in Cancer Initiation, Progression, and Therapy Resistance,” Theranostics 10, no. 19 (2020): 8721–8743, 10.7150/thno.41648. PMC7392012 32754274

[120] C. Li , Y. Zhou , R. Wei , et al., “Glycolytic Regulation of Intestinal Stem Cell Self‐Renewal and Differentiation,” Cellular and Molecular Gastroenterology and Hepatology 15, no. 4 (2023): 931–947, 10.1016/j.jcmgh.2022.12.012. PMC9971054 36584817

[121] L. Pan , F. Feng , J. Wu , et al., “Demethylzeylasteral Targets Lactate by Inhibiting Histone Lactylation to Suppress the Tumourigenicity of Liver Cancer Stem Cells,” Pharmacological Research 181 (2022): 106270, 10.1016/j.phrs.2022.106270. 35605812

[122] J. Deng , Y. Li , L. Yin , et al., “Histone Lactylation Enhances GCLC Expression and Thus Promotes Chemoresistance of Colorectal Cancer Stem Cells Through Inhibiting Ferroptosis,” Cell Death & Disease 16, no. 1 (2025): 193, 10.1038/s41419-025-07498-z. PMC11926133 40113760

[123] Q. Zhang , Z. Han , J. H. Liu , et al., “Hypoxia Facilitates Stemness of Colon Cancer Cells via Histone Lactylation,” Biochimica et Biophysica Acta ‐ Molecular Basis of Disease 1871, no. 8 (2025): 167993, 10.1016/j.bbadis.2025.167993. 40716503

[124] F. Feng , J. Wu , Q. Chi , et al., “Lactylome Analysis Unveils Lactylation‐Dependent Mechanisms of Stemness Remodeling in the Liver Cancer Stem Cells,” Advanced Science 11, no. 38 (2024): e2405975, 10.1002/advs.202405975. PMC11481176 39099416

[125] W. Huimin , W. Xin , Y. Shan , et al., “Lactate Promotes the Epithelial‐Mesenchymal Transition of Liver Cancer Cells via TWIST1 Lactylation,” Experimental Cell Research 447, no. 1 (2025): 114474, 10.1016/j.yexcr.2025.114474. 39993459

[126] M. Najafi , B. Farhood , and K. Mortezaee , “Cancer Stem Cells (CSCs) in Cancer Progression and Therapy,” Journal of Cellular Physiology 234, no. 6 (2019): 8381–8395, 10.1002/jcp.27740. 30417375

[127] Z. Miao , X. Zhao , and X. Liu , “Hypoxia Induced β‐Catenin Lactylation Promotes the Cell Proliferation and Stemness of Colorectal Cancer Through the Wnt Signaling Pathway,” Experimental Cell Research 422, no. 1 (2023): 113439, 10.1016/j.yexcr.2022.113439. 36464122

[128] F. Yan , Y. Teng , X. Li , et al., “Hypoxia Promotes Non‐Small Cell Lung Cancer Cell Stemness, Migration, and Invasion via Promoting Glycolysis by Lactylation of SOX9,” Cancer Biology & Therapy 25, no. 1 (2024): 2304161, 10.1080/15384047.2024.2304161. PMC10793688 38226837

[129] J. Li , Z. Chen , M. Jin , et al., “Histone H4K12 Lactylation Promotes Malignancy Progression in Triple‐Negative Breast Cancer Through SLFN5 Downregulation,” Cellular Signalling 124 (2024): 111468, 10.1016/j.cellsig.2024.111468. 39395526

[130] C. Chen , A. Lin , J. Zhao , et al., “Beyond Metabolism: Exploring the Regulatory and Therapeutic Implications of Lactate and Lactylation in Cancer‐Regulated Cell Death,” Cell Death & Disease 17, no. 1 (2026): 184, 10.1038/s41419-026-08410-z. PMC12876995 41571623

[131] B. Zhao , Y. Liu , X. Shen , et al., “Unraveling the Role of Lactylation in Cell Death and Correlative Diseases: A Comprehensive Review,” Frontiers in Cell and Development Biology 14 (2026): 1874067, 10.3389/fcell.2026.1874067. PMC13368751 42459826

[132] Y. Chen , Q. Yan , S. Ruan , et al., “GCLM Lactylation Mediated by ACAT2 Promotes Ferroptosis Resistance in KRAS(G12D)‐Mutant Cancer,” Cell Reports 44, no. 6 (2025): 115774, 10.1016/j.celrep.2025.115774. 40503938

[133] K. Zhang , L. Guo , X. Li , Y. Hu , and N. Luo , “Cancer‐Associated Fibroblasts Promote Doxorubicin Resistance in Triple‐Negative Breast Cancer Through Enhancing ZFP64 Histone Lactylation to Regulate Ferroptosis,” Journal of Translational Medicine 23, no. 1 (2025): 247, 10.1186/s12967-025-06246-3. PMC11871786 40022222

[134] L. Jiang , C. Li , P. Teng , et al., “SIRT7‐Mediated ACSL3 Delactylation Suppresses Colorectal Cancer Progression by Orchestrating Tumor‐Associated Macrophage Ferroptosis and Polarization,” Cell Death & Disease 17 (2026): 805, 10.1038/s41419-026-09007-2. PMC13582799 42380090

[135] D. Wei , M. Zhang , T. Lei , and Q. Hu , “Lactylation in Colorectal Cancer: Regulatory Networks, Functional Mechanisms, and Clinical Translational Potential,” International Journal of Molecular Sciences 27, no. 10 (2026): 4480, 10.3390/ijms27104480. PMC13207785 42196462

[136] J. F. Zhu , D. P. Guo , H. N. Lv , Z. Y. Liang , J. Song , and W. Zeng , “Histone Lactylation‐Mediated Up‐Regulation of IGF2BP2 Enhances Ferroptosis Resistance via Nrf2 in Colorectal Cancer,” Clinical and Translational Medicine 15, no. 12 (2025): e70551, 10.1002/ctm2.70551. PMC12706171 41399129

[137] S. Qu , B. Feng , M. Xing , et al., “PRMT5 K240lac Confers Ferroptosis Resistance via ALKBH5/SLC7A11 Axis in Colorectal Cancer,” Oncogene 44, no. 32 (2025): 2814–2830, 10.1038/s41388-025-03457-2. 40447754

[138] D. Cai , G. C. Zhong , Z. Zhao , et al., “L‐Lactate‐Driven PSMD14 Lactylation and Stabilization Promote Lactate Production and Ferroptosis Resistance via ENO1 in Intrahepatic Cholangiocarcinoma,” Cancer Letters 646 (2026): 218394, 10.1016/j.canlet.2026.218394. 41786282

[139] Y. Sun , X. Duan , B. Zhang , et al., “GLUT3 Drives Paclitaxel Resistance in Peritoneal Metastatic Gastric Cancer by Promoting H3K18 Lactylation‐Mediated MAPKAP1 Transcription to Suppress Ferroptosis,” International Journal of Biological Sciences 22, no. 9 (2026): 4826–4846, 10.7150/ijbs.130059. PMC13182558 42157923

[140] X. Yang , C. Guo , Y. Lu , et al., “Lactylation of Beta‐Catenin Promotes Non‐Small Cell Lung Cancer by Inhibiting Ferroptosis Through TCF4‐Mediated GPX4 Transcription,” Journal of Translational Medicine 24, no. 1 (2026): 2, 10.1186/s12967-026-08247-2. PMC13343740 42135726

[141] M. Gao , S. Wang , Z. Huang , T. Wang , and Y. Yu , “H3K18la Facilitates TRA2A‐Mediated Alternative Splicing of STIL, Suppressing Ferroptosis and Cisplatin Treatment Sensitivity in Ovarian Cancer,” Cancer Research and Treatment 58 (2025): 1428–1448, 10.4143/crt.2025.752. PMC13680085 40907573

[142] W. D. Yang , M. R. Lu , Q. Shen , et al., “Lactylated NAT10 Contributes to Elesclomol‐Triggered Cuproptosis via the NAT10/ac4C‐DLAT‐mRNA/DLAT Positive Feedback Loop in CRC,” Proceedings of the National Academy of Sciences of the United States of America 123, no. 19 (2026): e2501185123, 10.1073/pnas.2501185123. PMC13168536 42085165

[143] M. Jia , X. Yue , W. Sun , et al., “ULK1‐Mediated Metabolic Reprogramming Regulates Vps34 Lipid Kinase Activity by Its Lactylation,” Science Advances 9, no. 22 (2023): eadg4993, 10.1126/sciadv.adg4993. PMC10413652 37267363

[144] W. Sun , M. Jia , Y. Feng , and X. Cheng , “Lactate Is a Bridge Linking Glycolysis and Autophagy Through Lactylation,” Autophagy 19 (2023): 3240–3241, 10.1080/15548627.2023.2246356. PMC10621282 37565742

[145] W. Li , C. Zhou , L. Yu , et al., “Tumor‐Derived Lactate Promotes Resistance to Bevacizumab Treatment by Facilitating Autophagy Enhancer Protein RUBCNL Expression Through Histone H3 Lysine 18 Lactylation (H3K18la) in Colorectal Cancer,” Autophagy 20 (2023): 1–17, 10.1080/15548627.2023.2249762. PMC10761097 37615625

[146] Y. Huang , G. Luo , K. Peng , et al., “Lactylation Stabilizes TFEB to Elevate Autophagy and Lysosomal Activity,” Journal of Cell Biology 223, no. 11 (2024): e202308099, 10.1083/jcb.202308099. PMC11354204 39196068

[147] H. Jin , P. Wu , C. Lv , et al., “Mannose Inhibits PKM2 Lactylation to Induce Pyroptosis in Bladder Cancer and Activate Antitumour Immune Responses,” Communications Biology 8, no. 1 (2025): 689, 10.1038/s42003-025-08130-8. PMC12045973 40312519

[148] Y. Chen , J. Wu , L. Zhai , et al., “Metabolic Regulation of Homologous Recombination Repair by MRE11 Lactylation,” Cell 187, no. 2 (2024): 294–311, 10.1016/j.cell.2023.11.022. PMC11725302 38128537

[149] J. Li , L. Feng , L. Zhang , et al., “Saikosaponin D Mitigates Radioresistance in Triple‐Negative Breast Cancer by Inducing MRE11 De‐Lactylation via HIF1α/HDAC5 Pathway,” Theranostics 15, no. 17 (2025): 8935–8951, 10.7150/thno.113517. PMC12439262 40963916

[150] X. He , Y. Li , J. Li , et al., “HDAC2‐Mediated METTL3 Delactylation Promotes DNA Damage Repair and Chemotherapy Resistance in Triple‐Negative Breast Cancer,” Advanced Science 12, no. 14 (2025): e2413121, 10.1002/advs.202413121. PMC11984901 39950833

[151] F. Li , H. Zhang , Y. Huang , et al., “Single‐Cell Transcriptome Analysis Reveals the Association Between Histone Lactylation and Cisplatin Resistance in Bladder Cancer,” Drug Resistance Updates 73 (2024): 101059, 10.1016/j.drup.2024.101059. 38295753

[152] J. Zhuang , L. Shen , M. Li , et al., “Cancer‐Associated Fibroblast‐Derived miR‐146a‐5p Generates a Niche That Promotes Bladder Cancer Stemness and Chemoresistance,” Cancer Research 83, no. 10 (2023): 1611–1627, 10.1158/0008-5472.Can-22-2213. 36939397

[153] J. He , W. Li , S. Wang , et al., “Cancer Associated Fibroblasts‐Derived Lactate Induces Oxaliplatin Treatment Resistance by Promoting Cancer Stemness via ANTXR1 Lactylation in Colorectal Cancer,” Cancer Letters 631 (2025): 217917, 10.1016/j.canlet.2025.217917. 40683418

[154] F. Ren , X. Pang , F. Jin , N. Luan , H. Guo , and L. Zhu , “Integration of scRNA‐Seq and Bulk RNA‐Seq to Reveal the Association and Potential Molecular Mechanisms of Metabolic Reprogramming Regulated by Lactylation and Chemotherapy Resistance in Ovarian Cancer,” Frontiers in Immunology 16 (2025): 1513806, 10.3389/fimmu.2025.1513806. PMC11907005 40093000

[155] H. Hong , X. Chen , H. Wang , X. Gu , Y. Yuan , and Z. Zhang , “Global Profiling of Protein Lysine Lactylation and Potential Target Modified Protein Analysis in Hepatocellular Carcinoma,” Proteomics 23, no. 9 (2023): e2200432, 10.1002/pmic.202200432. 36625413

[156] K. Niu , Z. Chen , M. Li , et al., “NSUN2 Lactylation Drives Cancer Cell Resistance to Ferroptosis Through Enhancing GCLC‐Dependent Glutathione Synthesis,” Redox Biology 79 (2025): 103479, 10.1016/j.redox.2024.103479. PMC11750563 39742570

[157] B. Chen , Y. Deng , Y. Hong , et al., “Metabolic Recoding of NSUN2‐Mediated m(5)C Modification Promotes the Progression of Colorectal Cancer via the NSUN2/YBX1/m(5)C‐ENO1 Positive Feedback Loop,” Advanced Science 11, no. 28 (2024): e2309840, 10.1002/advs.202309840. PMC11267267 38769664

[158] L. Yang , X. Wang , J. Liu , et al., “Prognostic and Tumor Microenvironmental Feature of Clear Cell Renal Cell Carcinoma Revealed by m6A and Lactylation Modification‐Related Genes,” Frontiers in Immunology 14 (2023): 1225023, 10.3389/fimmu.2023.1225023. PMC10450969 37638005

[159] Z. Zhao , Z. Zhang , Q. Cai , et al., “Lactylation Increases the Stability of RBM15 to Drives m6A Modification in Non‐Small‐Cell Lung Cancer Cells,” FASEB Journal 39, no. 6 (2025): e70493, 10.1096/fj.202500020RR. 40135634

[160] J. Sun , Y. Shi , K. Yu , et al., “Hypoxia‐Induced Histone Lactylation Drives Cisplatin Resistance in Bladder Cancer by Promoting RBM15‐Dependent m(6)A Methylation of IGFBP3,” Research (Washington, D.C.) 8 (2025): 970, 10.34133/research.0970. PMC12586852 41199784

[161] X. Hou , Z. Hong , H. Zeng , et al., “Lactylation in Cancer Biology: Unlocking New Avenues for Research and Therapy,” Cancer Commun (Lond) 45, no. 11 (2025): 1367–1406, 10.1002/cac2.70054. PMC12629867 40831053

[162] C. Fang , S. Zhou , K. Yu , et al., “Lactate Metabolism and Lactylation in Cancer: From Pathogenesis to Therapeutic Advances,” Signal Transduction and Targeted Therapy 11, no. 1 (2026): 2672, 10.1038/s41392-026-02672-x. PMC13190781 42161914

[163] W. Zhang , M. Xia , J. Li , et al., “Warburg Effect and Lactylation in Cancer: Mechanisms for Chemoresistance,” Molecular Medicine 31, no. 1 (2025): 146, 10.1186/s10020-025-01205-6. PMC12016192 40264038

[164] X. Wang , J. Chen , B. Wang , et al., “Impact of Lactylation on the Pathogenesis of Cancer and Its Clinical Application Potential (Review),” Molecular Medicine Reports 32, no. 6 (2025): 1–17, 10.3892/mmr.2025.13702. PMC12516806 41041837

[165] Y. Sui , Z. Shen , Z. Wang , J. Feng , and G. Zhou , “Lactylation in Cancer: Metabolic Mechanism and Therapeutic Strategies,” Cell Death Discovery 11, no. 1 (2025): 68, 10.1038/s41420-025-02349-4. PMC11842571 39979245

[166] Z. Liu , A. Li , Z. Ma , et al., “Lactate Metabolism and Protein Lactylation in Cancer,” Molecular Biomedicine 7, no. 1 (2026): 15, 10.1186/s43556-026-00417-4. PMC12946641 41746580

[167] D. Li , K. Wang , G. Lou , et al., “Navigating the Role of Protein Lactylation in Prostate Cancer and Its Implications for Immunotherapy,” Journal of Cancer 16, no. 8 (2025): 2706–2719, 10.7150/jca.114137. PMC12171000 40535811

[168] S. Tan , W. Zhang , Y. Ding , et al., “The Glycolysis‐Lactylation Axis: A Metabolic‐Epigenetic Driver of Immunosuppression and Therapy Resistance in Cancer,” Frontiers in Immunology 17 (2026): 1772701, 10.3389/fimmu.2026.1772701. PMC13120921 42058190

[169] P. Wu , J. Zhang , Z. Jiang , et al., “Lactate‐Lactylation in Tumor Angiogenesis and Progression: Mechanisms, Biomarker Potential, and Therapeutic Implications,” Biomarker Research 14, no. 1 (2026): 21, 10.1186/s40364-026-00898-z. PMC12857134 41593811

[170] X. Yu , J. Yang , J. Xu , et al., “Histone Lactylation: From Tumor Lactate Metabolism to Epigenetic Regulation,” International Journal of Biological Sciences 20, no. 5 (2024): 1833–1854, 10.7150/ijbs.91492. PMC10929197 38481814

[171] L. Zhao , H. Cui , Y. Li , Y. Ye , and Z. Shen , “Metabolite to Modifier: Lactate and Lactylation in the Evolution of Tumors,” MedComm 6, no. 10 (2025): e70413, 10.1002/mco2.70413. PMC12497687 41059490

[172] J. Li , C. Miao , H. Guo , et al., “Lactylation in Cancer: Molecular Mechanisms and Advances in Clinical Study,” Molecular Cancer 25, no. 1 (2026): 1, 10.1186/s12943-026-02573-1. PMC12951931 41566389

[173] Y. He , Y. Huang , P. Peng , Q. Yan , and L. Ran , “Lactate and Lactylation in Gastrointestinal Cancer: Current Progress and Perspectives (Review),” Oncology Reports 53, no. 1 (2025): 6, 10.3892/or.2024.8839. PMC11574708 39513579

[174] I. Spinello , E. Saulle , M. T. Quaranta , et al., “The Small‐Molecule Compound AC‐73 Targeting CD147 Inhibits Leukemic Cell Proliferation, Induces Autophagy and Increases the Chemotherapeutic Sensitivity of Acute Myeloid Leukemia Cells,” Haematologica 104, no. 5 (2019): 973–985, 10.3324/haematol.2018.199661. PMC6518905 30467201

[175] H. Rho and N. Hay , “Protein Lactylation in Cancer: Mechanisms and Potential Therapeutic Implications,” Experimental & Molecular Medicine 57, no. 3 (2025): 545–553, 10.1038/s12276-025-01410-7. PMC11958728 40128358

[176] W. Zhang , G. Huang , W. Tang , et al., “Lactylation‐Driven Therapeutic Resistance in Cancer: Mechanisms and Therapeutic Opportunities,” Genes & Diseases 13, no. 4 (2026): 101935, 10.1016/j.gendis.2025.101935. PMC13068659 41971017

[177] T. Li , H. Gu , C. Liu , et al., “Unveiling Lactylation: A Novel Frontier in Cancer Stemness and Therapy,” Advanced Science 13, no. 46 (2026): e21985, 10.1002/advs.202521985. PMC13393273 42489278

[178] A. Llibre , S. Kucuk , A. Gope , M. Certo , and C. Mauro , “Lactate: A Key Regulator of the Immune Response,” Immunity 58, no. 3 (2025): 535–554, 10.1016/j.immuni.2025.02.008. 40073846

[179] Z. N. Hao , X. P. Tan , Q. Zhang , J. Li , R. Xia , and Z. Ma , “Lactate and Lactylation: Dual Regulators of T‐Cell‐Mediated Tumor Immunity and Immunotherapy,” Biomolecules 14, no. 12 (2024): 1646, 10.3390/biom14121646. PMC11674224 39766353

[180] X. Ji and L. Xia , “Lactate and Lactylation in Cancer: Drivers of Immune Suppression and Microenvironmental Reprogramming,” Experimental Hematology & Oncology 14, no. 1 (2025): 128, 10.1186/s40164-025-00719-3. PMC12570437 41152975

[181] A. Naik and J. Decock , “Lactate Metabolism and Immune Modulation in Breast Cancer: A Focused Review on Triple Negative Breast Tumors,” Frontiers in Oncology 10 (2020): 598626, 10.3389/fonc.2020.598626. PMC7725706 33324565

[182] E. Kocianova , V. Piatrikova , and T. Golias , “Revisiting the Warburg Effect With Focus on Lactate,” Cancers 14, no. 24 (2022): 6028, 10.3390/cancers14246028. PMC9776395 36551514

[183] X. Lv , Y. Lv , and X. Dai , “Lactate, Histone Lactylation and Cancer Hallmarks,” Expert Reviews in Molecular Medicine 25 (2023): e7, 10.1017/erm.2022.42. 36621008

[184] A. Pereira‐Nunes , J. Afonso , S. Granja , and F. Baltazar , “Lactate and Lactate Transporters as Key Players in the Maintenance of the Warburg Effect,” Advances in Experimental Medicine and Biology 1219 (2020): 51–74, 10.1007/978-3-030-34025-4_3. 32130693

[185] Y. Yu , X. Huang , C. Liang , and P. Zhang , “Evodiamine Impairs HIF1A Histone Lactylation to Inhibit Sema3A‐Mediated Angiogenesis and PD‐L1 by Inducing Ferroptosis in Prostate Cancer,” European Journal of Pharmacology 957 (2023): 176007, 10.1016/j.ejphar.2023.176007. 37611839

[186] H. Yang , X. Zou , S. Yang , A. Zhang , N. Li , and Z. Ma , “Identification of Lactylation Related Model to Predict Prognostic, Tumor Infiltrating Immunocytes and Response of Immunotherapy in Gastric Cancer,” Frontiers in Immunology 14 (2023): 1149989, 10.3389/fimmu.2023.1149989. PMC10020516 36936929

[187] Z. Cheng , H. Huang , M. Li , X. Liang , Y. Tan , and Y. Chen , “Lactylation‐Related Gene Signature Effectively Predicts Prognosis and Treatment Responsiveness in Hepatocellular Carcinoma,” Pharmaceuticals (Basel) 16, no. 5 (2023): 6050644, 10.3390/ph16050644. PMC10221268 37242427

[188] H. Huang , K. Chen , Y. Zhu , et al., “A Multi‐Dimensional Approach to Unravel the Intricacies of Lactylation Related Signature for Prognostic and Therapeutic Insight in Colorectal Cancer,” Journal of Translational Medicine 22, no. 1 (2024): 211, 10.1186/s12967-024-04955-9. PMC10900655 38419085

[189] Y. Jiao , F. Ji , L. Hou , Y. Lv , and J. Zhang , “Lactylation‐Related Gene Signature for Prognostic Prediction and Immune Infiltration Analysis in Breast Cancer,” Heliyon 10, no. 3 (2024): e24777, 10.1016/j.heliyon.2024.e24777. PMC10838739 38318076

[190] X. Wang , X. Liu , R. Xiao , et al., “Histone Lactylation Dynamics: Unlocking the Triad of Metabolism, Epigenetics, and Immune Regulation in Metastatic Cascade of Pancreatic Cancer,” Cancer Letters 598 (2024): 217117, 10.1016/j.canlet.2024.217117. 39019144

[191] L. Yu , C. Jing , S. Zhuang , L. Ji , and L. Jiang , “A Novel Lactylation‐Related Gene Signature for Effectively Distinguishing and Predicting the Prognosis of Ovarian Cancer,” Translational Cancer Research 13, no. 5 (2024): 2497–2508, 10.21037/tcr-24-319. PMC11170528 38881917

[192] X. Zhang , Y. Li , and Y. Chen , “Development of a Comprehensive Gene Signature Linking Hypoxia, Glycolysis, Lactylation, and Metabolomic Insights in Gastric Cancer Through the Integration of Bulk and Single‐Cell RNA‐Seq Data,” Biomedicine 11, no. 11 (2023): 2948, 10.3390/biomedicines11112948. PMC10669360 38001949

[193] T. Peng , F. Sun , J. C. Yang , et al., “Novel Lactylation‐Related Signature to Predict Prognosis for Pancreatic Adenocarcinoma,” World Journal of Gastroenterology 30, no. 19 (2024): 2575–2602, 10.3748/wjg.v30.i19.2575. PMC11135411 38817665

[194] J. Pan , J. Zhang , J. Lin , Y. Cai , and Z. Zhao , “Constructing Lactylation‐Related Genes Prognostic Model to Effectively Predict the Disease‐Free Survival and Treatment Responsiveness in Prostate Cancer Based on Machine Learning,” Frontiers in Genetics 15 (2024): 1343140, 10.3389/fgene.2024.1343140. PMC10985269 38566813

[195] M. Gao , M. Wang , S. Zhou , et al., “Machine Learning‐Based Prognostic Model of Lactylation‐Related Genes for Predicting Prognosis and Immune Infiltration in Patients With Lung Adenocarcinoma,” Cancer Cell International 24, no. 1 (2024): 400, 10.1186/s12935-024-03592-y. PMC11656871 39696439

[196] Z. Wu , H. Wu , Y. Dai , et al., “A Pan‐Cancer Multi‐Omics Analysis of Lactylation Genes Associated With Tumor Microenvironment and Cancer Development,” Heliyon 10, no. 5 (2024): e27465, 10.1016/j.heliyon.2024.e27465. PMC10923869 38463768

